# Global Weak Rigidity of the Gauss–Codazzi–Ricci Equations and Isometric Immersions of Riemannian Manifolds with Lower Regularity

**DOI:** 10.1007/s12220-017-9893-1

**Published:** 2017-08-18

**Authors:** Gui-Qiang G. Chen, Siran Li

**Affiliations:** Mathematical Institute, University of Oxford, Oxford, OX2 6GG UK

**Keywords:** Weak rigidity, Global, Intrinsic, Gauss–Codazzi–Ricci equations, Riemannian manifolds, Isometric immersion, Isometric embedding, Lower regularity, Weak convergence, Approximate solutions, Geometric div-curl lemma, div-curl structure, Cartan formalism, Riemann curvature tensor, Primary: 53C24, 53C42, 53C21, 53C45, 57R42, 35M30, 35B35, 58A15, 58J10, Secondary: 57R40, 58A14, 58A17, 58A05, 58K30, 58Z05

## Abstract

We are concerned with the global weak rigidity of the Gauss–Codazzi–Ricci (GCR) equations on Riemannian manifolds and the corresponding isometric immersions of Riemannian manifolds into the Euclidean spaces. We develop a unified intrinsic approach to establish the global weak rigidity of both the GCR equations and isometric immersions of the Riemannian manifolds, independent of the local coordinates, and provide further insights of the previous local results and arguments. The critical case has also been analyzed. To achieve this, we first reformulate the GCR equations with div-curl structure intrinsically on Riemannian manifolds and develop a global, intrinsic version of the div-curl lemma and other nonlinear techniques to tackle the global weak rigidity on manifolds. In particular, a general functional-analytic compensated compactness theorem on Banach spaces has been established, which includes the intrinsic div-curl lemma on Riemannian manifolds as a special case. The equivalence of global isometric immersions, the Cartan formalism, and the GCR equations on the Riemannian manifolds with lower regularity is established. We also prove a new weak rigidity result along the way, pertaining to the Cartan formalism, for Riemannian manifolds with lower regularity, and extend the weak rigidity results for Riemannian manifolds with corresponding different metrics.

## Introduction

We are concerned with the global weak rigidity of the Gauss–Codazzi–Ricci (GCR) equations on Riemannian manifolds and the corresponding global weak rigidity of isometric immersions of the Riemannian manifolds into the Euclidean spaces. The problem of isometric immersions of Riemannian manifolds into the Euclidean spaces has been of considerable interest in the development of differential geometry, which has also led to important developments of new ideas and methods in nonlinear analysis and partial differential equations (PDEs) (*cf.* [24, 37–39, 50]). On the other hand, the GCR equations are a fundamental system of nonlinear PDEs in differential geometry (*cf.* [4, 5, 17, 21, 22, 36, 43]). In particular, the GCR equations serve as the compatibility conditions to ensure the existence of isometric immersions. Therefore, it is important to understand the global, intrinsic behavior of this nonlinear system on Riemannian manifolds for solving the isometric immersion problems and other important geometric problems, including the global weak rigidity of the GCR equations and isometric immersions. In general, the Gauss–Codazzi–Ricci system has no type, neither purely hyperbolic nor purely elliptic.

The weak rigidity problem for isometric immersions is to decide, for a given sequence of isometric immersions of an *n*-dimensional manifold with a $W^{1,p}$ metric whose second fundamental forms and normal connections are uniformly bounded in $L^p_\mathrm{loc}, p>n$, whether its weak limit is still an isometric immersion with the same $W^{1,p}_\mathrm{loc}$ metric. This rigidity problem has its motivation both from geometric analysis and nonlinear elasticity: The existence of isometric immersions of Riemannian manifolds with lower regularity corresponds naturally to the realization of elastic bodies with lower regularity in the physical space. See Ciarlet–Gratie–Mardare [9], Mardare [30], Szopos [44], and the references cited therein.

The local weak rigidity of the GCR equations with lower regularity has been analyzed in Chen–Slemrod–Wang [7, 8], in which the div-curl structure of the GCR equations in local coordinates has first been observed so that compensated compactness ideas, especially the div-curl lemma (*cf.* Murat [34] and Tartar [45]), can be employed in the local coordinates. One of the advantages of these techniques is their independence of the type of PDEs—hence independent of the sign of curvatures in the setting of isometric immersions of the Riemannian manifolds. The key results in [7, 8] are the weak rigidity of solutions to the GCR equations, which is known to be equivalent to the existence of local isometric immersions in the $C^\infty$ category as a classical result (*cf.* [24, 47]). However, such an equivalence for Riemannian manifolds with lower regularity (i.e., $W^{1,p}_\mathrm{loc}$ metric) is not a direct consequence of the aforementioned classical results. This problem has been treated recently in [9, 30–32] from the point of view of nonlinear elasticity.

In this paper, we analyze the global weak rigidity of both the GCR equations and isometric immersions—via a new approach, independent of local coordinates. Instead of writing the GCR equations in the local coordinates, we formulate the GCR equations *intrinsically* on Riemannian manifolds and develop a global, intrinsic version of compensated compactness and other nonlinear techniques to tackle the global weak rigidity on manifolds. Our aim is to develop a unified intrinsic approach to establish the global weak rigidity of the GCR equations and isometric immersions, and provide further insights of the results and arguments in [8, 30–32, 44] and the references cited therein. We also establish a new weak rigidity result along the way, pertaining to the Cartan formalism, for Riemannian manifolds with lower regularity, and extend the weak rigidity results for Riemannian manifolds with corresponding different (unfixed) metrics.

This paper is organized as follows: In Sect. 2, we start with some geometric notations and present some basic facts about isometric immersions and the GCR equations on Riemannian manifolds for subsequent developments. In Sect. 3, we first formulate and prove a general abstract compensated compactness theorem on Banach spaces in the framework of functional analysis. As a direct corollary, we obtain a global, intrinsic version of the div-curl lemma, which has been also further extended to a more general version. These serve as a basic tool for subsequent sections. In Sect. 4, we give a geometric proof for the global weak rigidity of the GCR equations on Riemannian manifolds. The formulation and proof in this section are independent of the local coordinates of the manifolds, and are based on the geometric div-curl structure of the GCR equations. In Sect. 5, the equivalence of global isometric immersions, the Cartan formalism, and the GCR equations for simply connected *n*-dimensional manifolds with $W^{1,p}_\mathrm{loc}$ metric, $p>n$, is established. Then, in Sect. 6, we analyze the weak rigidity for the critical case $n=2$ and $p=2$. In particular, we show the weak rigidity of the GCR equations when the codimension is 1. Finally, in Sect. 7, we first provide a proof of the weak rigidity of the Cartan formalism, which gives an alternative intrinsic proof of the main result in Sect. 4, and then extend the weak rigidity results to the more general case such that the underlying metrics of manifolds are allowed to be a strongly convergent sequence in $W^{1, p}, p>n$. To keep the paper self-contained, in the appendix, we provide a proof for a general version of the intrinsic div-curl lemma, Theorem 3.4, on Riemannian manifolds.

## Geometric Notations, Isometric Immersions, and the GCR Equations

In this section, we start with some geometric notations about manifolds and vector bundles for self-containedness, and then present some basic facts about isometric immersions and the GCR equations on Riemannian manifolds for subsequent developments.

### Notations: Manifolds and Vector Bundles

Throughout this paper, we denote (*M*, *g*) as an *n*-dimensional Riemannian manifold. By definition, *M* is a second-countable, Hausdorff topological space with an atlas of local charts $\mathcal {A}=\{(U_\alpha , \phi _\alpha )\,: \,\alpha \in \mathcal {I}\}$ such that each $U_\alpha \subset M$ is an open subset, $\phi _\alpha : U_\alpha \mapsto \phi _\alpha (U_\alpha ) \subset \mathbb {R}^n$ is a homeomorphism, and the transition functions $\phi _\alpha \circ \phi _\beta ^{-1}$ between the overlapping charts $U_\alpha$ and $U_\beta$ have required regularity.

If each $\phi _\alpha \circ \phi _\beta ^{-1}$ can be chosen to have positive definite Jacobian determinant almost everywhere, then *M* is said to be orientable. Moreover, *M* is simply connected if its fundamental group is trivial, i.e., each loop on *M* can be continuously deformed to a point. The Riemannian metric *g* on *M* is given by a field of inner products. That is, at each point $P\in M$, $g(P): \Gamma (TM)\times \Gamma (TM)\mapsto \mathbb {R}$ is an inner product denoted by

$$\begin{aligned} g(P)(X,Y)=\langle X, Y\rangle \qquad \,\, \hbox {for any}\,\,\ X,Y\in \Gamma (TM), \end{aligned}$$

where *g* varies with respect to *P*, $\Gamma (TM)$ denotes the space of vector fields on *M* with required regularity, and we have suppressed the dependence on *P* when using $\langle \cdot , \cdot \rangle$ to denote the inner product associated with the metric. Throughout this paper, we always denote by $\Gamma$ the space of sections of given vector bundles with required Sobolev regularity, which needs not be smooth or analytic, in order to be more suitable for the applications to the realization problem (*cf.* Sect. 5).

Given (*M*, *g*), an affine connection on *M* (more precisely, on *TM*) is a bilinear map $\nabla : \Gamma (TM)\times \Gamma (TM)\mapsto \Gamma (TM)$ such that, for any $f: M\mapsto \mathbb {R}$ with required regularity, and any $X,Y,Z\in \Gamma (TM)$,

$$\begin{aligned} \nabla _{fX} Y = f\nabla _X Y, \qquad \nabla _X (fY) = f\nabla _XY + X(f)Y, \end{aligned}$$

where *X*(*f*) is the directional derivative of *f* in the direction of *X*, namely, vector *X* is identified as the corresponding first-order differential operator.

We say that $\nabla$ is compatible with metric *g* if

$$\begin{aligned} Xg(Y,Z)=g(\nabla _XY, Z) + g(Y,\nabla _XZ), \end{aligned}$$

and $\nabla$ is torsion-free if

$$\begin{aligned} \nabla _XY-\nabla _YX =[X,Y], \end{aligned}$$

where the Lie bracket is defined by $[X,Y]=XY-YX$. There exists a unique compatible, torsion-free affine connection $\nabla$ on *M*, known as the Levi–Civita connection, where the bilinear map $\nabla$ is also called the covariant derivative. As a basic example, consider $(\bar{M},\bar{g})=(\mathbb {R}^m, g_0)$ with the Euclidean metric $g_0=\delta _{ij}$, i.e., the dot product, whose Levi–Civita connection $\bar{\nabla }$ is given by $\bar{\nabla }_XY := XY$.

Given a manifold *M*, we say that (*E*, *M*, *F*) is a vector bundle of degree $k\in \mathbb {N}$ over *M* if there is a surjection $\pi : E \mapsto M$ such that, for any $P \in M$, there exists a local neighborhood $U \subset M$ containing *P* so that there is a diffeomorphism $\psi _U:\pi ^{-1}(U) \mapsto U \times F$ with $\mathrm{pr}_1\circ \psi _U = \pi$ on $\pi ^{-1}(U)$, where map $\mathrm{pr}_1$ is the projection map onto the first coordinate, *E* and *F* are also differentiable manifolds, and $F \simeq \mathbb {R}^k$ (vector space isomorphism). In this bundle, *E* is called the total space, *F* is the fiber, *M* is the base manifold, $\pi$ is the projection of the bundle, and $\psi _U$ is termed as a *local trivialization*. For simplicity, we also say that *E* *is a vector bundle over* *M*.

If $E_1$ and $E_2$ are both vector bundles over *M*, we can take the direct sum and the quotient of the bundles, by taking the vector space direct sum and quotient of the fibers. Also, for a vector bundle $(E,M,F=\mathbb {R}^k)$ with projection $\pi$, the space of smooth sections is defined by $\Gamma (E):=\{s\in C^\infty (M;E)\,: \,\pi \circ s=\mathrm{id}_M\}$. We can define the affine connection $\nabla ^E: \Gamma (TM)\times \Gamma (E) \mapsto \Gamma (E)$, by linearity and the Leibniz rule. For our purpose, $\nabla ^E$ is required to restrict to the Levi–Civita connection on *M*.

As a primary example, consider $E=TM$, the tangent bundle over *M*. Then $\pi$ is the projection onto the base point in *M*, and $\Gamma (TM)$ is the space of smooth vector fields, agreeing with the previous notation. Moreover, $\nabla ^{TM}$ is precisely the Levi–Civita connection. Another example is the cotangent bundle $T^*M$ over *M*, whose fibers are the dual vector spaces of the fibers of *TM*.

Our next example is crucial to this paper. Consider $E_1=T^*M\otimes T^*M\otimes \cdots \otimes T^*M$, the tensor product of *q*-copies of $T^*M$, for $q=0,1,2,\ldots$. This is the (covariant) *q*-tensor algebra over *M*, which can be viewed as *q*-linear maps on *TM*. Now let $E_2\subset E_1$ be the subspace of all the alternating *q*-tensors on *TM*, i.e., the *q*-linear maps that change sign when switching any pair of its indices $\{i,j\}\subset \{1,\ldots ,q\}$. By convention, we write $E_2=:\bigwedge ^q T^*M$, known as the alternating *q*-algebra. Moreover, the sections are $\Omega ^q(M):=\Gamma (\bigwedge ^q T^*M)$, known as the differential *q*-forms. A general element $\alpha \in \Omega ^q(M)$ is written as a linear combination of alternating forms $\xi _1 \wedge \cdots \wedge \xi _q$, where $\{\xi _j\}_{j=1}^q$ is a *q*-tuple of linearly independent differential 1-form on *M*. If $\dim (M)=n$, $\Omega ^q(M)= \{0\}$ for $q \ge n+1$. Thus, we always restrict to $0 \le q \le n$.

On the space of differential forms, we recall four important operations. The first is the exterior derivative $$d:\Omega ^q(M)\mapsto \Omega ^{q+1}(M)$$, which is linear and satisfies $$d^2=0$$. The second is the Hodge star $$*: \bigwedge ^q T^*M\mapsto \bigwedge ^{n-q}T^*M$$, which can also be regarded as $$*: \Omega ^q(M)\mapsto \Omega ^{n-q}(M)$$. It is an isomorphism of vector bundles, which satisfies $$**=(-1)^{q(n-q)}$$ whenever *M* is orientable. The third is a natural *product* on differential forms: For $$\alpha \in \Omega ^q(M)$$ and $$\beta \in \Omega ^r(M)$$, we can define the wedge product $$\alpha \wedge \beta \in \Omega ^{q+r}(M)$$. The fourth is the covariant derivative $$\nabla : \Omega ^q(M) \mapsto \Omega ^{q+1}(M)$$: For $$\alpha \in \Omega ^q(M)$$, define $$\nabla \alpha \in \Omega ^{q+1}(M)$$ via the Leibniz rule:

2.1 $$\begin{aligned}&(\nabla \alpha )(X, Y_1, \ldots , Y_q) \equiv \nabla _X\alpha (Y_1, \ldots , Y_q) \nonumber \\&\quad :=X \big (\alpha (Y_1, \ldots , Y_q)\big ) - \alpha (\nabla _X Y_1, \ldots , Y_q) - \cdots - \alpha (Y_1, \ldots , \nabla _X Y_q) \end{aligned}$$

for any $$X, Y_1, \ldots , Y_q \in \Gamma (TM)$$.

There is a natural isomorphism between *TM* and $T^*M$, by identifying canonically each fiber of *TM* with its dual. It induces a canonical isomorphism $\sharp : \Omega ^1(M) \mapsto \Gamma (TM)$, for which we write $\sharp ^{-1}=:\flat$. Note that, if a vector field and its corresponding 1-form in local coordinates are written by the Einstein summation convention, $\sharp$ (or $\flat$) amounts to raising (or lowering) the indices of the coefficients. Clearly, they extend to the isomorphisms between $T^*M \otimes \cdots \otimes T^*M$ (i.e., covariant tensors) and $TM \otimes \cdots \otimes TM$ (i.e., contravariant tensors).

For instance, consider the covariant derivative $\nabla : \Omega ^q(M)\mapsto \Omega ^{q+1}(M)$ defined above. For $q=1$ and $\alpha \in \Omega ^1(M)$, we set $X:=\alpha ^\sharp \in \Gamma (TM)$. Since $\nabla _YX \in \Gamma (TM)$ for any $Y \in \Gamma (TM)$, we can view $\nabla X = \nabla \alpha ^\sharp \in \Gamma (TM \otimes TM)$, i.e., $(\nabla \alpha ^\sharp )^\flat \in \Omega ^2(M)$. This example shows that, via the identifications $\sharp$ and $\flat$, the covariant derivative $\nabla$ on $\Omega ^q(M)$ generalizes the definition of the Levi–Civita connection.

Now let us briefly review how differential *n*-forms can be integrated on *n*-dimensional manifolds. On any orientable *n*-dimensional Riemannian manifold (*M*, *g*), there is a natural *n*-form $dV_g \in \Omega ^n(M)$, called the volume form, which satisfies $*1=dV_g$ and $*dV_g = 1$. Let $\mathcal {A}=\{(U_\alpha ,\phi _\alpha ): \alpha \in \mathcal {I}\}$ be an atlas for *M* as before. By the basic manifold theory, there exists a locally finite $C^\infty$-partition $\{\rho _\alpha : \alpha \in \mathcal {I}\}$ of unity subordinate to $\mathcal {A}$, i.e., $\sum _{\alpha \in \mathcal {I}} \rho _\alpha =1$, $0 \le \rho _\alpha \le 1$, and $supp (\rho _\alpha ) \Subset U_\alpha$. We define the integration of $\omega \in \Omega ^n(M)$ over *M* by

2.2 $$\begin{aligned} \int _M\omega :=\sum _{\alpha \in \mathcal {I}} \int _{\mathbb {R}^n} \rho _\alpha ((\phi _\alpha ^{-1})^*\omega ) \chi _{\phi _\alpha (U_\alpha )} \sqrt{|g|}\, \mathrm{d}x_1\cdots \mathrm{d}x_n, \end{aligned}$$

where $\phi ^*$ denotes the pullback of a tensor under $\phi$ with required regularity on *M*, and $|g| := \det (g_{ij})$. The integration of function $\phi$ on *M* is defined as the integration of its Hodge dual, i.e., $\int _M \phi := \int _M\phi \, \mathrm{d}V_g$.

We now define the Sobolev spaces $W^{k,p}(M; \bigwedge ^q T^*M)$ on an *n*-dimensional Riemannian manifold (*M*, *g*), which generalize $W^{k,p}(\mathbb {R}^n)$ for $k\in \mathbb {Z}$ and $1\le p \le \infty$. First, for differential *q*-forms $\alpha ,\beta \in \Omega ^q(M)$, *g* on *M* defines an inner product $g(\alpha ,\beta ) =\langle \alpha ,\beta \rangle$ by

2.3 $$\begin{aligned} \langle \alpha ,\beta \rangle \, dV_g := \alpha \wedge *\beta , \end{aligned}$$

which is an equality of *n*-forms on *M*. Then, for alternating contravariant *q*-tensor fields *T* and *S*, set $\langle T,S\rangle := \langle T^\flat , S^\flat \rangle$, which is consistent with the previous notations. Next, the $L^p$-norm of $\alpha \in \Omega ^q(M)$ is defined as

2.4 $$\begin{aligned} \Vert \alpha \Vert _{L^p} := \Big (\int _M \big [*(\alpha \wedge *\alpha )\big ]^{\frac{p}{2}} \,\mathrm{d}V_g\Big )^{\frac{1}{p}} = \Big (\int _M \langle \alpha ,\alpha \rangle ^{\frac{p}{2}}\,\mathrm{d}V_g\Big )^{\frac{1}{p}}. \end{aligned}$$

Moreover, for $\alpha \in \Omega ^q(M)$, set

2.5 $$\begin{aligned} \Vert \alpha \Vert _{W^{k,p}}:=\left( \sum _{j=0}^k \Vert \nabla ^j \alpha \Vert ^p_{L^p} \right) ^{\frac{1}{p}} = \left( \sum _{j=0}^k \Vert \underbrace{\nabla \circ \cdots \circ \nabla }_{j \text { times}} \alpha \Vert ^p_{L^p} \right) ^{\frac{1}{p}}. \end{aligned}$$

We denote by $W^{k,p}(M; \bigwedge ^qT^*M)$ the completion of the space of compactly supported *q*-forms with respect to $\Vert \cdot \Vert _{W^{k,p}}$. Notice that, for any contravariant tensor field *X*, the following holds:

$$\begin{aligned} \Vert X\Vert _{W^{k,p}}:=\Vert X^\flat \Vert _{W^{k,p}}. \end{aligned}$$

In fact, $W^{k,p}(M;E)$ can be defined for an arbitrary bundle *E*. Furthermore, we can define the $W^{k,p}$ connection $\nabla ^E$ on bundle *E*. This can be done since the moduli space of connections is an affine space modeled over the tensor algebra . We refer the reader to Jost [26] for the detailed construction. A key feature for this definition lies in its intrinsic nature, since the local coordinates on *M* are not needed to define $W^{k,p}(M;\bigwedge ^q T^*M)$. In particular, when $p=2$, we denote $H^{k}(M;\bigwedge ^q T^*M):=W^{k,2}(M;\bigwedge ^q T^*M)$ which are Hilbert spaces.

### Isometric Immersions

We are concerned with the isometric immersions of an *n*-dimensional manifold (*M*, *g*) into the Euclidean spaces. A map $f: (M,g)\hookrightarrow (\mathbb {R}^{n+k}, g_0)$ is an isometric immersion if the differential $df: TM \rightarrow T\mathbb {R}^{n+k}$ is everywhere injective, and

2.6 $$\begin{aligned} g_0(f(P))(df_P(X),df_P(Y)) = g(P) (X,Y) \end{aligned}$$

for every $P \in M$ and $X,Y\in \Gamma (TM)$. Since $g_0$ is the Euclidean dot product, Eq. (2.6) reads

2.7 $$\begin{aligned} \langle df_P(X), df_P(Y)\rangle = df_P(X) \cdot df_P(Y) = \langle X, Y \rangle . \end{aligned}$$

Then it is natural to ask whether, for any given (*M*, *g*), there is an isometric immersion *f* (or embedding, i.e., in addition, *f* is injective everywhere) into the Euclidean space $\mathbb {R}^{n+k}$. The existence of smooth isometric embeddings was established in Nash [38] in the large when the dimension of the target Euclidean space is high enough: For any (*M*, *g*), there exist a large enough *k* and a corresponding smooth isometric embedding $f: (M,g) \hookrightarrow (\mathbb {R}^{n+k}, g_0)$ (also see [23]). To achieve this, the problem was approached directly from Eq. (2.7), which is a first-order system of fully nonlinear PDEs, generically under-determined when *k* is large.

On the other hand, some progress has been made on the existence and regularity of immersions/embeddings of two-dimensional Riemannian manifolds $M^2$ into $\mathbb {R}^3$, with the minimal target dimension 3, the Janet dimension. In this setting, the problem can be reduced to a fully nonlinear Monge–Ampère equation, whose type is determined by the Gauss curvature *K* of *M*. For $K>0, K=0$, and $K<0$, the corresponding equation is elliptic, parabolic, and hyperbolic, respectively. The case of $K>0$ has a solution in the large due to Nirenberg [39], while the other two cases are more delicate, and are still widely open in the general setting; see Han–Hong [24].

### The Gauss–Codazzi–Ricci Equations

The isometric immersion problem can also be approached via the GCR equations as the compatibility conditions, instead of directly tackling Eq. (2.7) (*cf.* do Carmo [13]).

The GCR equations are derived from the orthogonal splitting of the tangent spaces along the isometric immersion $f: (M,g) \hookrightarrow (\mathbb {R}^{n+k}, g_0)$. Indeed, the tangent spaces satisfy $T_P\mathbb {R}^{n+k}\cong \mathbb {R}^{n+k}\cong T_PM \bigoplus T_PM^\perp$ for each $P \in M$, where $T_PM^\perp$ is the fiber of the *normal bundle* $TM^\perp$ at point *P*, and $TM^\perp$ is defined as the quotient vector bundle $T\mathbb {R}^{n+k}/TM$. Here and hereafter, we obey the widely adopted convention to identify *TM* with *T*(*fM*), that is, we view *f* as the inclusion map.

From now on, we will always use Latin letters $X,Y,Z,\ldots$ to denote tangential vector fields, i.e., elements in $\Gamma (TM)$, and Greek letters $\xi ,\eta ,\zeta ,\ldots$ to denote normal vector fields, i.e., elements in $\Gamma (TM^\perp )$.

The Levi–Civita connection $\bar{\nabla }$ on $\mathbb {R}^{n+k}$ is the trivial flat connection given by

2.8 $$\begin{aligned} \bar{\nabla }_V W:= VW = V^i(\partial _i W^j)\partial _j \qquad \,\, \text { for } V = V^i\partial _i, W=W^i\partial _i \in \Gamma (T\mathbb {R}^{n+k}), \end{aligned}$$

where we have used the Einstein summation convention. In other words, the covariant derivative corresponding to $\bar{\nabla }$ is just the usual derivative in Euclidean spaces. It is crucial for the study of isometric immersions that the projection of $\bar{\nabla }$ onto *TM* coincides with the Levi–Civita connection on *M* which is denoted by $\nabla$ in the sequel, and its projection onto $TM^\perp$ defines an affine connection on the normal bundle which is written as $\nabla ^\perp$; see §6 in [13]. Throughout this paper, we use notation $\bar{\nabla }_V W$ instead of *VW* to emphasize that $\{\nabla , \nabla ^\perp \}$ both come from the orthogonal splitting of $\bar{\nabla }$. We also adopt the convention that the geometric quantities with an overhead bar are associated with the *total space* $\mathbb {R}^{n+k}$ (as introduced in Sect. 1), while the quantities with superscript $\perp$ are associated with the normal bundle $TM^\perp$. Also, for any $X,Y\in \Gamma (TM)$, the vector field $\bar{\nabla }_XY \in T\mathbb {R}^{n+k}$ is well defined. Thus, we can define a symmetric bilinear form $B: \Gamma (TM)\times \Gamma (TM)\mapsto \Gamma (TM^\perp ) \nonumber$, known as the second fundamental form, by

2.9 $$\begin{aligned} B(X,Y) := \bar{\nabla }_XY - \nabla _XY. \end{aligned}$$

By a slightly abusive notation, we may view $B: \Gamma (TM)\times \Gamma (TM)\times \Gamma (TM^\perp )\mapsto \mathbb {R}$ as

$$\begin{aligned} B(X,Y,\xi ):= \langle B(X,Y),\xi \rangle \qquad \,\,\,\, \hbox {for }X,Y\in \Gamma (TM)\hbox { and }\xi \in \Gamma (TM^\perp ). \end{aligned}$$

Furthermore, for $\xi \in \Gamma (TM^\perp )$, define $S_\xi : \Gamma (TM)\mapsto \Gamma (TM)$, the shape operator (sometimes also called the second fundamental form), by

2.10 $$\begin{aligned} S_\xi X:= -\bar{\nabla }_X\xi + \nabla ^\perp _X \xi . \end{aligned}$$

Equivalently, it can be defined by contracting *B*:

2.11 $$\begin{aligned} B(X,Y,\xi ) =: \langle S_\xi X, Y\rangle . \end{aligned}$$

In addition, the Riemann curvature tensor on *M* is a rank-4 tensor $R: \Gamma (TM)\times \Gamma (TM)\times \Gamma (TM)\times \Gamma (TM)\mapsto \mathbb {R}$, defined by

2.12 $$\begin{aligned} R(X,Y,Z,W):=\langle \nabla _X\nabla _Y Z, W\rangle -\langle \nabla _Y\nabla _X Z, W\rangle + \langle \nabla _{[X,Y]}Z, W\rangle . \end{aligned}$$

Notice that the last two coordinates (*Z*, *W*) do not enter the definition of *R* in an essential way. In fact, for any vector bundle *E* over *M* with affine connection $\nabla ^E$, we can define the Riemann curvature on *E* as $R^E:\Gamma (TM)\times \Gamma (TM)\times \Gamma (E) \times \Gamma (E) \mapsto \mathbb {R}$ by

2.13 $$\begin{aligned} R^E(X,Y,s_1,s_2):= & {} \langle [\nabla ^E_X, \nabla ^E_Y] s_1, s_2 \rangle - \langle \nabla ^E_{[X,Y]}s_1, s_2 \rangle \end{aligned}$$

for $X,Y\in \Gamma (TM)$ and $s_1,s_2 \in \Gamma (E).$ For our purpose, we consider three bundles over *M*: $(TM, \nabla )$, $(TM^\perp , \nabla ^\perp )$, and $(T\mathbb {R}^{n+k},\bar{\nabla })$. We denote their Riemann curvatures by $R, R^\perp$, and $\bar{R}$, respectively.

With the geometric notations above, we are now at the stage of introducing the GCR equations. The GCR equations express the flatness of the Euclidean space $(\mathbb {R}^{n+k}, g_0)$, i.e., $\bar{R}=0$. We take any $X,Y \in \Gamma (TM)$ and sections $s_1,s_2\in \Gamma (E)$ in

2.14 $$\begin{aligned} \bar{R}(X,Y, s_1, s_2) = 0. \end{aligned}$$

Owing to the split: $T\mathbb {R}^{n+k}\simeq TM \bigoplus TM^\perp$ (at least locally), we can take $(s_1, s_2)$ to be one of the three combinations: (tangential, tangential), (tangential, normal), and (normal, normal). The resulting equations are named after Gauss, Codazzi, and Ricci, respectively.

#### Theorem 2.1

(GCR Equations) Assume that $f: (M,g) \hookrightarrow (\mathbb {R}^{n+k}, g_0)$ is an isometric immersion. Then the following Gauss–Codazzi–Ricci equations are satisfied:

2.15 $$\begin{aligned}&\langle B(Y,W), B(X,Z) \rangle - \langle B(X,W), B(Y,Z)\rangle =R(X,Y,Z,W), \end{aligned}$$

2.16 $$\begin{aligned}&\bar{\nabla }_Y B(X,Z) = \bar{\nabla }_X B(Y,Z), \end{aligned}$$

2.17 $$\begin{aligned}&\langle [S_\eta , S_\xi ]X, Y\rangle = R^\perp (X,Y,\eta ,\xi ). \end{aligned}$$

In Theorem 2.1, we have expressed the GCR equations in the most compact form. Nevertheless, to analyze the weak rigidity, it is helpful to rewrite the Codazzi and Ricci equations in a less concise manner.

#### Theorem 2.2

The following equations are equivalent to the GCR system:

2.18 $$\begin{aligned} \langle B(X,W), B(Y,Z)\rangle - \langle B(Y,W), B(X,Z) \rangle= & {} -R(X,Y,Z,W), \end{aligned}$$

2.19 $$\begin{aligned} XB(Y,Z,\eta ) - YB(X,Z,\eta )= & {} B([X,Y],Z,\eta ) \nonumber \\&- B(X,\nabla _YZ,\eta ) - B(X,Z,\nabla ^\perp _Y\eta )\nonumber \\&+ B(Y,\nabla _XZ,\eta ) + B(Y,Z,\nabla ^\perp _X\eta ), \nonumber \\ \end{aligned}$$

2.20 $$\begin{aligned} X\langle \nabla ^\perp _Y \xi ,\eta \rangle - Y \langle \nabla ^\perp _X\xi ,\eta \rangle= & {} \langle \nabla ^\perp _{[X,Y]}\xi ,\eta \rangle - \langle \nabla ^\perp _X \xi , \nabla ^\perp _Y \eta \rangle \nonumber \\&+ \langle \nabla ^\perp _Y\xi , \nabla ^\perp _X \eta \rangle +B(\bar{\nabla }_X \xi - \nabla ^\perp _X \xi , Y, \eta )\nonumber \\&- B(\bar{\nabla }_X \eta - \nabla ^\perp _X \eta , Y, \xi ) \end{aligned}$$

for any tangential vector fields $X,Y,Z,W\in \Gamma (TM)$ and any normal vector fields $\xi ,\eta \in \Gamma (TM^\perp )$.

#### Proof

The Gauss equation (2.18) takes the same form as in Theorem 2.1. For the Codazzi equation (2.19), using the Leibniz rule, we have

$$\begin{aligned} \bar{\nabla }_XB(Y,Z,\eta )&= X\langle B(Y,Z),\eta \rangle - \langle B(\nabla _XY, Z), \eta \rangle - \langle B(Y,\nabla _XZ), \eta \rangle \\&\qquad - \langle B(Y,Z), \nabla ^\perp _X\eta \rangle \\&= \langle \nabla _X^\perp B(Y,Z), \eta \rangle - \langle B(\nabla _XY, Z), \eta \rangle - \langle B(Y,\nabla _XZ), \eta \rangle , \end{aligned}$$

as well as the analogous expression for $\bar{\nabla }_YB(X,Z,\eta )$:

$$\begin{aligned} \bar{\nabla }_YB(X,Z,\eta ) = \langle \nabla _Y^\perp B(X,Z), \eta \rangle - \langle B(\nabla _YX, Z), \eta \rangle - \langle B(X,\nabla _YZ), \eta \rangle . \end{aligned}$$

Equating these two expressions by Eq. (2.16) and noticing that $\nabla _XY-\nabla _YX = [X,Y]$, we obtain Eq. (2.19).

For the Ricci equation (2.20), the right-hand side of Eq. (2.17) can be expanded as

$$\begin{aligned} R^\perp (X,Y,\eta ,\xi )&= \langle \nabla ^\perp _X\nabla ^\perp _Y\eta ,\xi \rangle - \langle \nabla ^\perp _Y \nabla ^\perp _X \eta ,\xi \rangle + \langle \nabla ^\perp _{[X,Y]}\eta , \xi \rangle \\&= X\langle \nabla ^\perp _Y\eta , \xi \rangle - \langle \nabla ^\perp _Y\eta , \nabla ^\perp _X\xi \rangle - Y\langle \nabla ^\perp _X\eta ,\xi \rangle + \langle \nabla ^\perp _X\eta ,\nabla ^\perp _Y\xi \rangle \\&\qquad + \langle \nabla ^\perp _{[X,Y]}\eta , \xi \rangle , \end{aligned}$$

by the definition of $R^\perp$ and the Leibniz rule. Moreover, the left-hand side of   Eq. (2.17) equals $\langle S_\eta S_\xi X, Y\rangle - \langle S_\xi S_\eta X, Y \rangle$, where

$$\begin{aligned} \langle S_\eta S_\xi X, Y\rangle&= B(S_\xi X, Y, \eta ) = -B(\bar{\nabla }_X\xi -\nabla ^\perp _X\xi , Y, \eta ), \end{aligned}$$

thanks to the definition of *S*; Similarly,

$$\begin{aligned} \langle S_\xi S_\eta X, Y\rangle = -B(\bar{\nabla }_X\eta -\nabla ^\perp _X\eta , Y, \xi ). \end{aligned}$$

Thus, we obtain Eq. (2.20). This completes the proof.

In the GCR equations in the form of either Theorem 2.1 or Theorem 2.2, we view $(B, \nabla ^\perp )$ as unknowns and *g* (hence *R*) as given. The GCR equations constitute a necessary condition for the existence of isometric immersions. Tenenblat [47] proved that, if everything is smooth, this is also sufficient for the local existence of isometric immersions.

From the point of view of PDEs, the three equations in Theorem 2.2 form a system of first-order nonlinear equations. The left-hand sides of Eqs. (2.19)–(2.20) can be regarded as the principal parts, while the nonlinear terms on the right-hand sides are of zero-th order. The nonlinear terms are quadratic in the form of $B\otimes B$, $\nabla ^\perp \otimes \nabla ^\perp$, and $B\otimes \nabla ^\perp$.

## Intrinsic Compensated Compactness Theorems on Riemannian Manifolds

In this section, we first formulate and prove a general abstract compensated compactness theorem in the framework of functional analysis (in Sect. 3.1). As a special case, it implies a global intrinsic version of the div-curl lemma on Riemannian manifolds, which generalizes the well-known classical versions in $\mathbb {R}^n$, first by Murat [34] and Tartar [45]. Such a geometric div-curl lemma, which is presented in Sect. 3.2, will serve as a basic tool in the subsequent development.

### General Compensated Compactness Theorem on Banach Spaces

Throughout this section, for a normed vector space *X* with dual space $X^*$, we use $\langle \cdot ,\cdot \rangle _X$ to denote the duality pairing of $(X,X^*)$. Let $\mathcal {H}$ be a Hilbert space over field $\mathbb {K}=\mathbb {R}\text { or } \mathbb {C}$ such that $\mathcal {H}=\mathcal {H}^*$. Let *Y* and *Z* be two Banach spaces over $\mathbb {K}$ with their dual spaces $Y^*$ and $Z^*$, respectively. In what follows, we consider two bounded linear operators $S:\mathcal {H}\mapsto Y$, $T:\mathcal {H}\mapsto Z$, and their adjoint operators $S^\dagger : Y^*\mapsto \mathcal {H}$ and $T^\dagger : Z^* \mapsto \mathcal {H}$, respectively.

Furthermore, the following conventional notations are adopted: For any normed vector spaces *X*, $X_1$, and $X_2$, we write $\{s^\epsilon \}\subset X$ for a sequence $\{s^\epsilon \}$ in *X* as a subset, and $X_1 \Subset X_2$ for a compact embedding between the normed vector spaces. We use $\Vert \cdot \Vert _X$ to denote the norm in *X*. Also, we use $\rightarrow$ to denote the strong convergence of sequences and $\rightharpoonup$ for the weak convergence. Furthermore, we denote the closed unit ball of space *X* by $\bar{B}_X:=\{x\in X: \Vert x\Vert \le 1\}$, the open unit ball by $B_X:=\{x\in X: \Vert x\Vert <1\}$. Moreover, for a linear operator $L: X_1\mapsto X_2$, its kernel is written as $\ker (L)\subset X_1$, and its range is denoted by $\text {ran}(L)\subset X_2$. Finally, for $X_1\subset X$ as a vector subspace, its *annihilator* is defined as $X_1^\perp :=\{f\in X^*:f(x)=0 \text { for all } x\in X_1\}$.

To formulate the compensated compactness theorem in the general functional-analytic framework, we first introduce the following two bounded linear operators: Define

$$\begin{aligned} {\left\{ \begin{array}{ll} S\oplus T: \mathcal {H}\mapsto Y\bigoplus Z,\\ \quad \,\, (S\oplus T) h:=(Sh,Th) \,\,\,\hbox {for }h\in \mathcal {H};\\ S^\dagger \vee T^\dagger : (Y\bigoplus Z)^*\cong Y^*\bigoplus Z^* \mapsto \mathcal {H}, \\ \quad \,\,(S^\dagger \vee T^\dagger )(a,b) := S^\dagger a + T^\dagger b \,\,\, \hbox {for }a\in Y^*\hbox { and }b\in Z^*. \end{array}\right. } \end{aligned}$$

Here and throughout, the Banach space $Y\bigoplus Z$ is endowed with norm $\Vert (y,z)\Vert _{Y\bigoplus Z}:=\Vert y\Vert _Y+\Vert z\Vert _Z$. Notice that, for any $a\in Y^*, b\in Z^*$, and $h\in \mathcal {H}$,

$$\begin{aligned} \langle h, (S^\dagger \vee T^\dagger )(a,b)\rangle \!= \!\langle h, S^\dagger a \!+\! T^\dagger b\rangle \!=\!\langle Sh, a \rangle _Y \!+\! \langle Th, b\rangle _Z \!=\! \langle (S\oplus T)h, (a,b)\rangle _{Y\bigoplus Z}. \end{aligned}$$

Thus, $S^\dagger \vee T^\dagger$ is in fact the adjoint operator of $S\oplus T$, namely $(S\oplus T)^\dagger = S^\dagger \vee T^\dagger$.

In the setting above, we are concerned with the following question:

#### Question

Let $\{u^\epsilon \}$, $\{v^\epsilon \}\Subset \mathcal {H}$ be two sequences so that there exist $\bar{u}, \bar{v}\in \mathcal {H}$ such that $u^\epsilon \rightharpoonup \bar{u}$ and $v^\epsilon \rightharpoonup \bar{v}$ in $\mathcal {H}$ as $\epsilon \rightarrow 0$. Under which conditions does the following hold:

$$\begin{aligned} \langle u^\epsilon , v^\epsilon \rangle _\mathcal {H}\rightarrow \langle \bar{u},\bar{v}\rangle _\mathcal {H}\qquad \text { as } \epsilon \rightarrow 0? \end{aligned}$$

The goal of this subsection is to provide a sufficient condition for the convergence $\langle u^\epsilon , v^\epsilon \rangle _\mathcal {H}\rightarrow \langle \bar{u},\bar{v}\rangle _\mathcal {H}$ as $\epsilon \rightarrow 0$. Roughly speaking, it requires the existence of a “nice” pair of bounded linear operators $S:\mathcal {H}\mapsto Y$ and $T:\mathcal {H}\mapsto Z$ such that *S* and *T* are orthogonal to each other, and $S\oplus T$ gains certain compactness/regularity. More precisely, we prove

#### Theorem 3.1

(General Compensated Compactness Theorem on Banach Spaces). Let $\mathcal {H}$ be a Hilbert space over $\mathbb {K}$, *Y* and *Z* be reflexive Banach spaces over $\mathbb {K}$, and let $S:\mathcal {H}\mapsto Y$ and $T:\mathcal {H}\mapsto Z$ be bounded linear operators satisfying

1. Orthogonality:
  3.1 $$\begin{aligned} S\circ T^\dagger = 0, \qquad T\circ S^\dagger =0; \end{aligned}$$
2. For some Hilbert space  so that $\mathcal {H}$ embeds compactly into , there exists a constant $C>0$ such that, for any $h\in \mathcal {H}$, 3.2

Assume that two sequences $\{u^\epsilon \}, \{v^\epsilon \}\subset \mathcal {H}$ satisfy the following conditions:

1. $u^\epsilon \rightharpoonup \bar{u}$ and $v^\epsilon \rightharpoonup \bar{v}$ in $\mathcal {H}$ as $\epsilon \rightarrow 0$;
2. $\{Su^\epsilon \}$ is pre-compact in *Y*, and $\{Tv^\epsilon \}$ is pre-compact in *Z*.

Then

$$\begin{aligned} \langle u^\epsilon , v^\epsilon \rangle _\mathcal {H}\rightarrow \langle \bar{u}, \bar{v}\rangle _\mathcal {H}\qquad \text { as } \epsilon \rightarrow 0. \end{aligned}$$

#### Proof

We divide the proof into eight steps.

**1.** In order to show that  has a finite-dimensional kernel, we consider the following subset of $\mathcal {H}$:where  is a compact embedding between the Hilbert spaces.

Suppose that  is compact. First notice that *j*(*E*) is the closed unit ball of $j[\ker (S\oplus T)]$, which is a Banach space, due to the closedness of the kernel. It then follows from the classical Riesz lemma that $j[\ker (S\oplus T)]$ is finite-dimensional. Since *j* is an embedding, we can conclude that $\dim \ker (S\oplus T)=\dim (j[\ker (S\oplus T)])<\infty$.

To prove the compactness of *j*(*E*), take any $h\in E$ and consider the following estimate deduced from condition (Op 2):3.3Hence, the boundedness of *E* in $\mathcal {H}$ and the compactness of  imply that *j*(*E*) is compact. Thus, the first step is complete.

**2.** We now show that $S\oplus T$ is a closed-ranged operator, i.e., $\text {ran}(S\oplus T)\subset Y\bigoplus Z$ is a closed subspace. To this end, we first prove the existence of a constant $\epsilon _0>0$ such that, for all $h\in \mathcal {H}$,3.4In fact, if the inequality were false, then, for any $\mu \in (0,1)$, we could find $\{h^\mu \}\subset [\ker (S\oplus T)]^\perp$ and  such that

$$\begin{aligned} \Vert (S\oplus T)h^\mu \Vert _{Y\bigoplus Z}\le \mu \end{aligned}$$

and

$$\begin{aligned} \mathrm{dist}\big (h^\mu , \ker (S\oplus T)\big )\ge \hat{\epsilon }_0 \end{aligned}$$

for some $\hat{\epsilon }_0>0$. Such a choice of $\{h^\mu \}$ is possible, owing to the finite-dimensionality of $S\oplus T$. Now, plugging $\{h^\mu \}$ into condition (Op 2), we haveIt follows from the compactness of *j* that $\{j(h^\mu )\}$ is pre-compact in . Let *j*(*h*) be a limit point of $\{j(h^\mu )\}$ in . Then one must have $h\in \ker (S\oplus T)$, in view of $\Vert (S\oplus T)h^\mu \Vert _{Y\bigoplus Z}\le \mu$. However, this contradicts the fact that $\{h^\mu \}$ were chosen to have a distance at least $\hat{\epsilon }_0$ from $\ker (S\oplus T)$. Therefore, we have established estimate (3.4).

To proceed, consider a sequence $\{h^\mu \}\subset \mathcal {H}$ such that $(S\oplus T)h^\mu \rightarrow w$ for some $w\in Y\bigoplus Z$. Our goal is to show that $w\in \text {ran}(S\oplus T)$. Indeed, by the projection theorem for Hilbert spaces, we can decompose $h^\mu =k^\mu +r^\mu$ with $k^\mu \in \ker (S\oplus T)$ and $r^\mu =[\ker (S\oplus T)]^\perp$ (which is a closed subspace of $\mathcal {H}$). Then estimate (3.4) gives3.5As a consequence, $\{j(r^\mu )\}$ is a Cauchy sequence in $j(\mathcal {H})$, which implies that there exists $j(r)\in j(\mathcal {H})$ such that $j(r^\mu )\rightarrow j(r)$ in $j(\mathcal {H})$ as $\mu \rightarrow 0$. Since *j* is an embedding, it follows that $r^\mu \rightarrow r$ in $\mathcal {H}$. Therefore, by the closed graph theorem of Banach spaces, $(S\oplus T)h^\mu =(S\oplus T)r^\mu \rightarrow (S\oplus T)r$. It now follows that $w=(S\oplus T)r$ so that the range of $S\oplus T$ is closed.

**3.** As an immediate corollary, we can obtain the following decomposition of $\mathcal {H}$:

3.6 $$\begin{aligned} \mathcal {H}= \ker (S\oplus T) \bigoplus \text {ran}(S^\dagger \vee T^\dagger ). \end{aligned}$$

Here, $\bigoplus$ denotes the topological direct sum of Banach spaces, and the direct summands are orthogonal as Hilbert spaces.

Indeed, by the projection theorem, $\mathcal {H}=\ker (S\oplus T)\bigoplus [\ker (S\oplus T)]^\perp$. On the other hand,

$$\begin{aligned}{}[\ker (S\oplus T)]^\perp =\overline{\text {ran}[(S\oplus T)^\dagger ]}=\overline{\text {ran}(S^\dagger \vee T^\dagger )}. \end{aligned}$$

We recall the closed range theorem in Banach spaces, which states that a bounded linear operator is closed-ranged if and only if its adjoint operator is closed-ranged (*cf.* [20]). Hence, we may deduce from the preceding equalities that

$$\begin{aligned}{}[\ker (S\oplus T)]^\perp =\text {ran}(S^\dagger \vee T^\dagger ). \end{aligned}$$

The decomposition in Eq. (3.6) now follows immediately.

With this decomposition, it now suffices to prove Theorem 3.1 for *surjective* operators *S* and *T*. Indeed, all the conditions in (Op 1)–(Op 2) and (Seq 1)–(Seq 2) continue to hold when $Y\bigoplus Z$ is replaced by $\text {ran}(S\oplus T)$, which has been proved to be a closed subspace of $Y\bigoplus Z$. Here we have used the fact that closed subspaces of reflexive Banach spaces are still reflexive. Therefore, in the sequel, we always assume $\text {ran}(S\oplus T)=Y\bigoplus Z$ without loss of generality.

**4.** Now, we introduce the following operator :3.7For this *generalized Laplacian*, we also prove that it has a finite-dimensional kernel, and its range is closed (in fact, surjective, in view of the reduction at the end of Step 3).

Indeed, we can find the range of  as follows:3.8where the decomposition in Eq. (3.6) has been used in the second equality.

On the other hand, notice that3.9In this expression, the first direct summand equals $\ker (S\oplus T)$, by a similar argument as in Eq. (3.8). For the second summand, a standard result in functional analysis gives $\ker (S^\dagger \vee T^\dagger ) = [\text {ran}(S\oplus T)]^\perp$, which is assumed to be $\{0\}$ at the end of Step 3. It follows that , which is finite-dimensional, by Step 1.

To summarize,  is a closed-ranged operator with a finite-dimensional kernel; without loss of generality, we may assume  to be surjective.

**5.** We now show a crucial result concerning :First, applying the open mapping theorem in Banach spaces to operator , we can find a constant $\delta >0$ and an element  such that .

From this inclusion, we can prove3.10In fact, for any $v_0 \in \delta \bar{B}_{Y\bigoplus Z}$, we can write $v_0$ as a convex combination of elements in $w_0+\delta B_{Y\bigoplus Z}$, e.g., $v_0=\frac{1}{2}\big ((v_0+w_0)+(v_0-w_0)\big )$. Observe that  is a convex set, as  is a bounded linear operator and $B_{Y^*\bigoplus Z^*}$ is convex. Since $w_0+\delta B_{Y\bigoplus Z}$ lies in , we conclude that . Thus, (3.10) now follows.

As a consequence, given any , there exists $\eta \in \bar{B}_{Y^*\bigoplus Z^*}$ such that . Define $\xi :=\frac{\Vert w\Vert }{\delta }\eta$, which yieldsNow, the proof for $(\clubsuit )$ is completed by choosing $M=\delta ^{-1}$.

**6.** Now, we employ (3.6) to decompose sequences $\{u^\epsilon \}$ and $\{v^\epsilon \}$, and the weak limits $\bar{u}$ and $\bar{v}$. In the sequel, we denote the canonical projection of $\mathcal {H}$ onto the first factor by $\pi _1:\mathcal {H}=\ker (S\oplus T)\bigoplus \text {ran}(S^\dagger \vee T^\dagger ) \mapsto \ker (S\oplus T)$. By Step 1, $\pi _1$ is a finite-rank (hence compact) operator.

Employing such a decomposition, we can write3.11for some $a,\tilde{a}, a^\epsilon ,\tilde{a}^\epsilon \in Y^*$ and $b,\tilde{b}, b^\epsilon , \tilde{b}^\epsilon \in Z^*$. Moreover, applying the orthogonality condition (Op 1), we haveSince $\{u^\epsilon \}$ and $\{v^\epsilon \}$ are weakly convergent by assumption (Seq 1), and $\pi _1$ is a compact operator, we obtain that $\langle \pi _1u^\epsilon , \pi _1v^\epsilon \rangle \rightarrow \langle \pi _1\bar{u}, \pi _1\bar{v}\rangle$. It thus remains to establish3.12In the next two steps, we prove (3.12).

**7.** The starting point is to observe that the left-hand side of (3.12) can be rewritten as follows:3.13On the other hand, let us apply *S* to $u^\epsilon$ and *T* to $v^\epsilon$ in (3.11). Using the definition of $\pi _1$ and the orthogonality condition (Op 1), we immediately find that3.14i.e., . As $\{Su^\epsilon \}\subset Y$ and $\{Tv^\epsilon \}\subset Z$ are pre-compact by (Seq 2), it suffices to show the boundedness of $\{(\tilde{a}^\epsilon , b^\epsilon )\}\subset Y^*\bigoplus Z^*$ to conclude (3.12). To see this point, assuming the boundedness, one can deduce thatFor $\text {I}^\epsilon$, since $\Vert (\tilde{a}^\epsilon ,b^\epsilon )\Vert _{Y^*\bigoplus Z^*}\le M$, we can use the pre-compactness of $\{Su^\epsilon \}\subset Y$ and $\{Tv^\epsilon \}\subset Z$ in assumption (Seq 1) to conclude that

$$\begin{aligned} \text {I}^\epsilon \le M \big \Vert \big (S(u^\epsilon -\bar{u}), T(v^\epsilon - \bar{v})\big ) \big \Vert _{Y\bigoplus Z} \rightarrow 0. \end{aligned}$$

For $$\text {II}^\epsilon $$, we need to invoke the reflexivity of *Y* and *Z*. As a Banach space is reflexive if and only if its dual space is reflexive, we know that $$Y^*\bigoplus Z^*$$ is reflexive. Then the bounded sequence $$\{(\tilde{a}^\epsilon , b^\epsilon )\}$$ is weakly pre-compact, by Theorem 3.31 in [20]. Moreover, Theorem 4.47 (Eberlein-S̆mulian theorem) in [20] implies the weak convergence of $$\{(\tilde{a}^\epsilon ,b^\epsilon )\}$$. Thus, viewing  as an element in $$(Y\bigoplus Z)^{**}$$, we conclude that $$\text {II}^\epsilon \rightarrow 0$$.

**8.** From the above arguments, it remains to prove the boundedness of $\{(\tilde{a}^\epsilon , b^\epsilon )\}\subset Y^*\bigoplus Z^*$. We further remark that $(\tilde{a}^\epsilon , b^\epsilon )$ can be chosen *modulo* . More precisely, it is enough to exhibit one particular representative $(\tilde{a}^\epsilon , b^\epsilon )$ in  such that $\Vert (\tilde{a}^\epsilon , b^\epsilon )\Vert _{Y^*\bigoplus Z^*}\le C$, where $C<\infty$ is independent of $\epsilon$. To see this, notice thatanalogous to Eqs. (3.13)–(3.14). Therefore, we have the freedom to choose a representative $(\tilde{a}^\epsilon ,b^\epsilon )$ in the co-set , without changing the expression on the left-hand side of (3.12).

For this purpose, let us invoke the key estimate $(\clubsuit )$ proved in Step 5 to find a constant $M\in (0, \infty )$ satisfying

$$\begin{aligned} \Vert (\tilde{a}^\epsilon , b^\epsilon )\Vert _{Y^*\bigoplus Z^*} \le M\Vert (Sv^\epsilon , Tu^\epsilon )\Vert _{Y\bigoplus Z}. \end{aligned}$$

Then it suffices to prove the uniform boundedness of $\{(Sv^\epsilon , Tu^\epsilon )\}$ in $Y\bigoplus Z$.

Indeed, as $u^\epsilon \rightharpoonup \bar{u}$ and $v^\epsilon \rightharpoonup \bar{v}$ according to assumption (Seq 1), we know that $\{u^\epsilon \}$ and $\{v^\epsilon \}$ are bounded in $\mathcal {H}$, by using the weak lower semi-continuity of $\Vert \cdot \Vert _\mathcal {H}$. By the reflexivity of Hilbert spaces (due to the Riesz representation theorem), $\{u^\epsilon \}$ and $\{v^\epsilon \}$ are weakly pre-compact in $\mathcal {H}$. Next, by standard results in functional analysis, the continuous linear operator $S\oplus T:\mathcal {H}\mapsto Y\bigoplus Z$ with respect to the strong topologies is also continuous when both $\mathcal {H}$ and $Y\bigoplus Z$ are endowed with the weak topologies (see [20] for details). As continuous mappings take pre-compact sets to pre-compact sets, $\{(Sv^\epsilon , Tu^\epsilon )\}$ is weakly pre-compact in $Y\bigoplus Z$; equivalently, we have established the uniform boundedness of $\{(Sv^\epsilon , Tu^\epsilon )\}$ in the strong topology of $Y\bigoplus Z$, because the Banach spaces *Y* and *Z* are reflexive.

Therefore, we have proved that

3.15 $$\begin{aligned} \Vert (\tilde{a}^\epsilon , b^\epsilon )\Vert _{Y^*\bigoplus Z^*} \le M \sup _{\epsilon >0}\Vert (Sv^\epsilon , Tu^\epsilon )\Vert _{Y\bigoplus Z}\le M'<\infty , \end{aligned}$$

from which the convergence in (3.12) follows. This completes the proof. $\square$

Let us close this subsection with two remarks on Theorem 3.1. First, in terms of the applications, *Y* and *Z* are usually Hilbert spaces $H^s=W^{s,2}$ for $s\in \mathbb {Z}$, or *S* and *T* are known to be Fredholm operators. In such cases, our arguments can be essentially simplified. Second, the reflexivity of *Y* and *Z* is necessary for Theorem 3.1 to hold.

#### Remark 3.1

If *Y* and *Z* are Hilbert spaces, then  is self-adjoint: . Thus, we can decompose3.16(cf. Proposition 7.32 in [20]) so that the closed-rangedness of  and the crucial estimate $(\clubsuit )$ are automatically verified. As a consequence, after establishing the finite-dimensionality of  as in the first half of Step 1, we can proceed to Step 5 in the proof of Theorem 3.1. On the other hand, if *S* and *T* are given *a priori* to have finite-dimensional kernels and co-kernels, then $S\oplus T$ is a Fredholm operator (whose range is automatically closed). Again, we can directly proceed to Step 5.

#### Remark 3.2

The assumption of reflexivity of *Y* and *Z* is indispensable. One counterexample for nonreflexive *Y* and *Z* is the “Fakir’s carpet” in Conti–Dolzmann–Müller [12] (a more extensive variant also appeared in DiPerna–Majda [16]). Consider the following vector fields on $\Omega :=(0,1)^3$:

3.17 $$\begin{aligned} u^\epsilon (x) = v^\epsilon (x) = \Big (\sqrt{m}\sum _{j=1}^m\chi _{\left[ \frac{j}{m}, \frac{j}{m}+\frac{1}{m^2}\right] }, 0, 0\Big )^\top \qquad \hbox {with }m:= \left\lfloor \frac{1}{\epsilon }\right\rfloor , \end{aligned}$$

operators $S=\mathrm{div}$, $T=\mathrm{curl}$, and spaces $\mathcal {H} = L^2(\Omega ; \mathbb {R}^3)$, $Y=W^{-1,1}(\Omega )$, and $Z=W^{-1,1}(\Omega ; \mathbb {R}^3)$. Then $u^\epsilon \rightharpoonup 0$ and $v^\epsilon \rightharpoonup 0$ in $\mathcal {H}$, but $\langle u^\epsilon , v^\epsilon \rangle \rightarrow 1$, as $\epsilon \rightarrow 0$, where $\langle \cdot ,\cdot \rangle$ denotes the $L^2$ inner product. On the other hand, for any test function $\psi \in W^{1,\infty }_0(\Omega )$ and any test vector field $\phi =(\phi ^1, \phi ^2, \phi ^3)^\top \in W^{1,\infty }_0(\Omega ; \mathbb {R}^3)$, we have

$$\begin{aligned} \Big |\int _\Omega Su^\epsilon (x)\psi (x)\,\mathrm{d}x\Big |&= \Big |\sqrt{m}\sum _{j=1}^m \int _{\frac{j}{m}}^{\frac{j}{m} + \frac{1}{m^2}} \frac{\partial \psi }{\partial x^1}(x)\,\mathrm{d}x\Big |\\&\le \Vert \psi \Vert _{W^{1,\infty }(\Omega )} \frac{1}{\sqrt{m}} \longrightarrow 0 \qquad \text { as } \epsilon \rightarrow 0, \end{aligned}$$

and similarly

$$\begin{aligned} \Big |\int _\Omega Tv^\epsilon (x)\cdot \phi (x)\,\mathrm{d}x\Big |&= \Big |\int _\Omega v^\epsilon (x) \cdot \mathrm{curl}\,\phi (x)\,\mathrm{d}x \Big |\\&= \Big |\sqrt{m}\sum _{j=1}^m \int _{\frac{j}{m}}^{\frac{j}{m} + \frac{1}{m^2}} \Big (\frac{\partial \phi ^3}{\partial x^2} - \frac{\partial \phi ^2}{\partial x^3}\Big ) \,\mathrm{d}x\Big |\\&\le \Vert \phi \Vert _{W^{1,\infty }(\Omega ; \mathbb {R}^3)}\frac{1}{\sqrt{m}} \longrightarrow 0 \qquad \text { as } \epsilon \rightarrow 0. \end{aligned}$$

Therefore, $Su^\epsilon \rightharpoonup 0$ in *Y* and $Tv^\epsilon \rightharpoonup 0$ in *Z*.

The failure of the weak continuity is related to the phenomenon of “concentration” in fluid mechanics, nonlinear elasticity, and calculus of variations; see Sect. 6 for further discussions.

### Intrinsic Div-Curl Lemma on Riemannian Manifolds

Now we adapt the general functional-analytic theorem, Theorem 3.1, to the geometric settings. Our aim is to obtain a global intrinsic div-curl lemma on Riemannian manifolds (Theorem 3.3), independent of local coordinates, which will be applied to analyze the global weak rigidity of isometric immersions of Riemannian manifolds in the subsequent development. Let us begin with the notions of several geometric quantities.

First of all, it is well known that the *divergence* of a vector field is globally defined for an arbitrary dimensional orientable manifold *M*:

3.18 $$\begin{aligned} \mathrm{div} X:= *d *(X^\flat ) \equiv *(\mathcal {L}_X dV_g) \qquad \,\,\,\hbox {for any }X\in \Gamma (TM), \end{aligned}$$

where $*$ is the Hodge star, $\mathcal {L}$ is the Lie derivative, and $dV_g$ is the volume form with respect to metric *g*. Geometrically, $\mathrm{div} X$ measures the change of volume in the direction of *X*. The *gradient* can also be defined in higher dimensions:

3.19 $$\begin{aligned} \mathrm{grad} f:= (df)^\sharp . \end{aligned}$$

The notion of *curl* is more subtle. Our ordinary definition for *curl*, if we require to be intrinsic, is only well defined on three-dimensional manifolds. This is because one needs to identify canonically $\Omega ^2(M)$ with $\Gamma (TM)$, which is only valid in three dimensions via the Hodge duality. Physically, $\mathrm{curl}(X)$ measures the rotation of the flow generated by *X* pointing to the rotation axis, where the direction of the rotation axis is unambiguous in three dimensions. For $X\in \Gamma (TM)$ with $\dim M=3$,

3.20 $$\begin{aligned} \mathrm{curl} X:= [*d (X^\flat )]^\sharp , \end{aligned}$$

where $\sharp \circ *$ identifies the 2-form $d X^\flat$ with the vector field. On an arbitrarily dimensional manifold *M*, we can define the *generalized curl* just as the 2-form, without pulling back to vector fields:

3.21 $$\begin{aligned} \mathrm{curl} X:= d(X^\flat ). \end{aligned}$$

#### Remark 3.3

The underlying reason for introducing the generalized curl is the algebra isomorphism $\bigwedge ^2(T^*M) \cong \mathfrak {so}(n)$, which is the Lie algebra of the special orthogonal group. Our $\mathrm{curl}(X)$ is just a field of anti-symmetric matrices which can be naturally interpreted as rotations, thanks to the structure of $\mathfrak {so}(n)$.

Next, we define $\delta : \Omega ^q(M) \mapsto \Omega ^{q-1}(M)$ to be the (formal) adjoint operator of *d* in the following sense:

3.22 $$\begin{aligned} \int _M \delta \alpha \wedge *\beta = \int _M \alpha \wedge *d\beta \qquad \,\, \text { for } \alpha \in \Omega ^q(M) \text { and } \beta \in \Omega ^{q-1}(M). \end{aligned}$$

To emphasize the dependence on metric *g*, it can also be expressed as

3.23 $$\begin{aligned} \int _M \langle \delta \alpha , \beta \rangle \,\mathrm{d} V_g = \int _M \langle \alpha ,d\beta \rangle \,\mathrm{d}V_g \qquad \text { for } \alpha \in \Omega ^q(M) \text { and } \beta \in \Omega ^{q-1}(M). \end{aligned}$$

Equivalently, we can define $$\delta := (-1)^{n(q+1)+1} *d*$$, where $$*$$ is the Hodge star. Hence, in the definition of divergence (3.18), $$\mathrm{div} X$$ is simply $$\delta X^\flat $$ modulo a sign. Therefore, we always regard $$\delta $$ as the *divergence* and *d* as the *curl*.

Moreover, the Laplace–Beltrami operator $\Delta : \Omega ^q(M)\mapsto \Omega ^q(M)$ on manifold *M* is defined for each $0\le q \le n$ as

3.24 $$\begin{aligned} \Delta :=d\circ \delta +\delta \circ d. \end{aligned}$$

Denote $\mathrm{Har}^q(M):=\ker (\Delta )$, the space of harmonic *q*-forms. Then $\alpha \in \mathrm{Har}^q(M)$ if and only if $d\alpha =0$ and $\delta \alpha =0$.

One fundamental result concerning the Laplace–Beltrami operator is the Hodge decomposition theorem (*cf.* Warner [48]):

#### Theorem 3.2

(Hodge Decomposition) Let *M* be a closed, orientable Riemannian manifold. For each integer *q* with $0\le q \le n$, the following orthogonal decomposition holds:

3.25 $$\begin{aligned} \Omega ^q(M)= \text {Har}^q(M)\bigoplus \mathrm{Im}(\Delta )= \text {Har}^q(M)\bigoplus d\Omega ^{q-1}(M) \bigoplus \delta \Omega ^{q+1}(M), \end{aligned}$$

where the orthogonality is taken with respect to the $L^2$ inner product on $\Omega ^q(M)$. Moreover, $\dim _\mathbb {R}(\text {Har}^q(M)) =\dim _\mathbb {R}(H_\mathrm{dR}^q(M;\mathbb {R}))< \infty$ for closed manifolds, where $H_\mathrm{dR}^q(M;\mathbb {R})$ is the $q^{\text {th}}$ *de Rham* cohomology group of *M*.

In view of Theorem 3.2, we can define the solution operator (i.e., the Green operator) to the Laplace–Beltrami on closed manifolds. Let $\pi _H: \Omega ^q(M)\mapsto \text {Har}^q(M)$ stand for the canonical projection. Then, for $\alpha \in \Omega ^q(M)$, we set

3.26 $$\begin{aligned} G(\alpha )= \Delta ^{-1}(\alpha -\pi _H\alpha ). \end{aligned}$$

It can be shown that, if *T* is a linear operator commuting with $\Delta$, then it also commutes with *G*. In particular, $d, \delta , \Delta$, and $*$ commute with *G*. Moreover, $\Delta$ and *G* are bounded linear operators on the Sobolev spaces. To be explicit, $\Delta : {W^{k+2,p}}(M;\bigwedge ^q T^*M) \mapsto W^{k,p}(M;\bigwedge ^q T^*M)$ and $G: W^{k,p}(M;\bigwedge ^q T^*M)\mapsto W^{k+2, p}(M;\bigwedge ^q T^*M)$ are continuous for each $k \in \mathbb {Z}$, $p\in (1,\infty )$, and $0 \le q\le n$.

#### Theorem 3.3

Let (*M*, *g*) be an *n*-dimensional Riemannian manifold. Let

$$\begin{aligned} \{\omega ^\epsilon \}, \{\tau ^\epsilon \} \subset L^2_\mathrm{loc}(M;\bigwedge ^q T^*M) \end{aligned}$$

be two families of differential *q*-forms, for $0\le q\le n$, such that

(i) $\omega ^\epsilon \rightharpoonup \overline{\omega }$ weakly in $L^2$, and $\tau ^\epsilon \rightharpoonup \overline{\tau }$ weakly in $L^2$;
(ii) there are compact subsets of the corresponding Sobolev spaces, $K_d$ and $K_\delta$, such that
  $$\begin{aligned} {\left\{ \begin{array}{ll} \{d\omega ^\epsilon \}\subset K_d \Subset H^{-1}_\mathrm{loc}(M;\bigwedge ^{q+1}T^*M),\\ \{\delta \tau ^\epsilon \}\subset K_\delta \Subset H^{-1}_\mathrm{loc}(M;\bigwedge ^{q-1}T^*M). \end{array}\right. } \end{aligned}$$

Then $\langle \omega ^\epsilon , \tau ^\epsilon \rangle$ converges to $\langle \overline{\omega }, \overline{\tau }\rangle$ in the sense of distributions:

$$\begin{aligned} \int _M \langle \omega ^\epsilon , \tau ^\epsilon \rangle \psi \, \mathrm{d}V_g \longrightarrow \int _M \langle \overline{\omega }, \overline{\tau }\rangle \psi \, \mathrm{d}V_g \qquad \hbox {for any }\psi \in C^\infty _{c}(M). \end{aligned}$$

#### Proof

First of all, we can reduce the statement of the theorem only for compact manifolds. Indeed, for any test function $\psi \in C^\infty _c(M)$, it suffices to assume that $\psi \ge 0$. Otherwise, we may decompose it as $\psi =\psi ^+ - \psi ^-$ and approximate $\psi ^\pm$ by $C^\infty$ nonnegative functions, respectively. Then, without loss of generality, we can always replace $(\omega ^\epsilon ,\tau ^\epsilon )$ by $(\sqrt{\psi }\omega ^\epsilon ,\sqrt{\psi }\tau ^\epsilon )$, and replace *M* by any compact submanifold of *M* containing the support of $\psi$. Thus we may drop the test function $\psi$ and the subscripts “loc” in the function spaces: More precisely, it suffices to take *M* as a closed Riemannian manifold and establish the convergence:

$$\begin{aligned} \int _M \langle \omega ^\epsilon , \tau ^\epsilon \rangle \, \mathrm{d}V_g \longrightarrow \int _M \langle \overline{\omega }, \overline{\tau }\rangle \, \mathrm{d}V_g \quad \hbox {as }\epsilon \rightarrow 0, \end{aligned}$$

under the assumptions that $\{\omega ^\epsilon \}, \{\tau ^\epsilon \} \subset L^2 (M;\bigwedge ^q T^*M)$, $\{d\omega ^\epsilon \}\subset K_d \Subset H^{-1}(M;\bigwedge ^{q+1}T^*M)$, and $\{\delta \tau ^\epsilon \}\subset K_\delta \Subset H^{-1}(M;\bigwedge ^{q-1}T^*M)$.

Now we are in the position of applying Theorem 3.1. For this purpose, we take $\mathcal {H}= L^2 (M;\bigwedge ^q T^*M)$, $Y=H^{-1}(M;\bigwedge ^{q+1}T^*M)$, $Z=H^{-1}(M;\bigwedge ^{q-1}T^*M)$, , $S=d$, and $T=\delta$. In this setting, $\{\omega ^\epsilon \}$ and $\{\tau ^\epsilon \}$ play the role of $\{u^\epsilon \}$ and $\{v^\epsilon \}$, respectively. Conditions (Seq 1)–(Seq 2) of Theorem 3.1 correspond precisely to conditions (i)–(ii), and condition (Op 1) of Theorem 3.1 is verified by the cohomology chain condition $d\circ d=0$ and $\delta \circ \delta =0$.

Thus, we are left with checking the estimate in (Op 2), i.e., for any $\alpha \in L^2(M;\bigwedge ^q T^*M)$, there exists a constant $C>0$ such that

3.27 $$\begin{aligned}&\Vert \alpha \Vert _{L^2(M;\bigwedge ^q T^*M)}\nonumber \\&\le \; C\Big (\Vert d\alpha \Vert _{H^{-1}(M;\bigwedge ^{q+1}T^*M)} +\Vert \delta \alpha \Vert _{H^{-1}(M;\bigwedge ^{q-1}T^*M)}+\Vert \alpha \Vert _{H^{-1}(M;\bigwedge ^{q}T^*M)}\Big ). \end{aligned}$$

To this end, we rely crucially on the Hodge decomposition theorem, Theorem 3.2, as well as the standard elliptic estimate for the Laplace–Beltrami operator in the following form:

3.28 $$\begin{aligned} \Vert \omega \Vert _{L^2(M;\bigwedge ^q T^*M)} \le C\Big (\Vert \Delta \omega \Vert _{H^{-2}(M;\bigwedge ^q T^*M)} + \Vert \omega \Vert _{H^{-2}(M;\bigwedge ^q T^*M)}\Big ) \end{aligned}$$

for arbitrary $$\omega \in L^2(M;\bigwedge ^q T^*M)$$.

Now let us prove (3.27). Given $\alpha \in L^2(M;\bigwedge ^q T^*M)$, we define $\beta :=G\alpha$, which is equivalent to the decomposition: $\alpha =\pi _H \alpha + \Delta \beta$. Applying the elliptic estimate (3.28) to $\omega :=\pi _H \alpha$, we have

$$\begin{aligned} \Vert \pi _H \alpha \Vert _{L^2(M;\bigwedge ^q T^*M)} \le C\Vert \pi _H\alpha \Vert _{H^{-2}(M;\bigwedge ^q T^*M)}, \end{aligned}$$

where $C>0$ is a constant, independent of $\alpha$. As $H^{-2}(M;\bigwedge ^q T^*M)$ is a Hilbert space and $\pi _H$ is a projection, the Pythagorean law gives that $\Vert \pi _H \alpha \Vert _{H^{-2}(M;\bigwedge ^q T^*M)} \le \Vert \alpha \Vert _{H^{-2}(M;\bigwedge ^q T^*M)}$. Thus, in view of the compact embedding: $H^{-1}(M;\bigwedge ^q T^*M)\hookrightarrow {H^{-2}(M;\bigwedge ^q T^*M)}$ due to the Rellich lemma, we conclude

3.29 $$\begin{aligned} \Vert \pi _H \alpha \Vert _{L^2(M;\bigwedge ^q T^*M)} \le C\Vert \alpha \Vert _{H^{-1}(M;\bigwedge ^q T^*M)}. \end{aligned}$$

Finally, we can bound $\Delta \beta$ in $L^2$. The bound follows from the following estimates:

3.30 $$\begin{aligned}&\Vert \Delta \beta \Vert _{L^2(M;\bigwedge ^q T^*M)}\nonumber \\&\quad =\, \Vert \Delta (G\alpha )\Vert _{L^2(M;\bigwedge ^q T^*M)}\nonumber \\&\quad =\, \Vert G(\Delta \alpha )\Vert _{L^2(M;\bigwedge ^q T^*M)} \nonumber \\&\quad \le \, C\Big (\Vert \Delta \alpha \Vert _{H^{-2}(M;\bigwedge ^q T^*M)} + \Vert G(\Delta \alpha )\Vert _{H^{-2}(M;\bigwedge ^q T^*M)} \Big )\nonumber \\&\quad \le \, C\Big (\Vert (d\delta +\delta d)\alpha \Vert _{H^{-2}(M;\bigwedge ^q T^*M)} + \Vert \alpha -\pi _H(\alpha )\Vert _{H^{-2}(M;\bigwedge ^q T^*M)} \Big )\nonumber \\&\quad \le \, C\Big (\Vert \delta \alpha \Vert _{H^{-1}(M;\bigwedge ^{q-1}T^*M)}+\Vert d\alpha \Vert _{H^{-1}(M;\bigwedge ^{q+1}T^*M)}+\Vert \alpha \Vert _{H^{-2}(M;\bigwedge ^q T^*M)} \Big )\nonumber \\&\quad \le \, C\Big (\Vert \delta \alpha \Vert _{H^{-1}(M;\bigwedge ^{q-1}T^*M)}+\Vert d\alpha \Vert _{H^{-1}(M;\bigwedge ^{q+1}T^*M)} +\Vert \alpha \Vert _{H^{-1}(M;\bigwedge ^{q}T^*M)}\Big ), \end{aligned}$$

where we have used the commutativity of *G* and $\Delta$ in the third line, the elliptic estimate in the fourth line with $\omega =G\alpha$ in (3.28), the definition of $\Delta$ and *G* in the fifth line, the Pythagorean theorem in the Hilbert space $H^{-2}(M;\bigwedge ^q T^*M)$ in the sixth line, and the Rellich lemma in the final line. Therefore, the estimate in (3.27) has been obtained, and the proof is now complete. $\square$

To conclude this section, we point out that some connections between the Hodge decomposition theorem and compensated compactness have been observed by Robbin–Rogers–Temple in [40] (also *cf.* Tartar [45]), and some generalized versions of the div-curl lemma have been obtained by Kozono–Yanagisawa [27]–[28], among others. Our general functional-analytic compensated compactness theorem, Theorem 3.1, further provides such a connection in the abstract form. In particular, our proof is essentially based upon the analysis of operator , which is an analogue of the Laplace–Beltrami operator $\Delta$.

In fact, a global intrinsic div-curl lemma, more general than Theorem 3.3, on Riemannian manifolds can also be established, which applies for the two sequences $\{\omega ^\epsilon \}$ and $\{\tau ^\epsilon \}$ lying in $L^r_{\mathrm{loc}}$ and $L^s_{\mathrm{loc}}$, where $1<r,s<\infty$ and $\frac{1}{r}+\frac{1}{s}=1$.

#### Theorem 3.4

Let (*M*, *g*) be an *n*-dimensional Riemannian manifold. Let $\{\omega ^\epsilon \} \subset L^r_\mathrm{loc}(M;\bigwedge ^q T^*M)$ and $\{\tau ^\epsilon \} \subset L^s_\mathrm{loc}(M;\bigwedge ^q T^*M)$ be two families of differential *q*-forms, for $0\le q\le n$, $1<r,s<\infty$, and $\frac{1}{r}+\frac{1}{s}=1$. Suppose that

(i) $\omega ^\epsilon \rightharpoonup \overline{\omega }$ weakly in $L^r$, and $\tau ^\epsilon \rightharpoonup \overline{\tau }$ weakly in $L^s$ as $\epsilon \rightarrow 0$;
(ii) There are compact subsets of the corresponding Sobolev spaces, $K_d$ and $K_\delta$, such that
  $$\begin{aligned} {\left\{ \begin{array}{ll} \{d\omega ^\epsilon \}\subset K_d \Subset W^{-1,r}_\mathrm{loc}(M;\bigwedge ^{p+1}T^*M),\\ \{\delta \tau ^\epsilon \}\subset K_\delta \Subset W^{-1,s}_\mathrm{loc}(M;\bigwedge ^{q-1}T^*M). \end{array}\right. } \end{aligned}$$

Then $\langle \omega ^\epsilon , \tau ^\epsilon \rangle$ converges to $\langle \overline{\omega }, \overline{\tau }\rangle$ in the sense of distributions: For any $\psi \in C^\infty _{c}(M)$,

$$\begin{aligned} \int _M \langle \omega ^\epsilon , \tau ^\epsilon \rangle \psi \, \mathrm{d}V_g \longrightarrow \int _M \langle \overline{\omega }, \overline{\tau }\rangle \psi \, \mathrm{d}V_g \qquad \hbox {as }\epsilon \rightarrow 0. \end{aligned}$$

To make this paper self-contained, we will present its proof in the appendix.

## Global Weak Rigidity of the Gauss–Codazzi–Ricci Equations on Riemannian Manifolds

In this section, we establish the global weak rigidity of the GCR equations. on Riemannian manifolds, independent of the local coordinates.

### Global Weak Rigidity Theorem

Our main result in this section is the following:

#### Theorem 4.1

(Global Weak Rigidity of the GCR Equations). Let (*M*, *g*) be an *n*-dimensional manifold with $W^{1,p}$ metric for $p > 2$. Let a sequence of operators $\{(B^\epsilon ,\nabla ^{\perp ,\epsilon })\}$ satisfy

(i) The tensor fields $B^\epsilon : \Gamma (TM)\times \Gamma (TM)\mapsto \Gamma (TM^\perp )$ and the affine connections $\nabla ^{\perp ,\epsilon }: \Gamma (TM)\times \Gamma (TM^\perp )\mapsto \Gamma (TM^\perp )$ are uniformly bounded in $L^p_\mathrm{loc}$ with
  4.1 $$\begin{aligned} \sup _{\epsilon >0} \big \{\Vert B^\epsilon \Vert _{L^p(K)} + \Vert \nabla ^{\perp ,\epsilon }\Vert _{L^p(K)} \big \} \le C_K \end{aligned}$$
  for a constant $C_K$ on any $K\Subset M$ compact subsets, independent of $\epsilon$;
(ii) $(B^\epsilon ,\nabla ^{\perp ,\epsilon })$ are solutions of the GCR equations in the distributional sense: For any $X,Y,Z,W \in \Gamma (TM)$ and $\eta ,\xi \in \Gamma (TM^\perp )$,
  4.2 $$\begin{aligned}&\langle B^\epsilon (X,W), B^\epsilon (Y,Z)\rangle -\langle B^\epsilon (Y,W), B^\epsilon (X,Z) \rangle =-R(X,Y,Z,W), \qquad \end{aligned}$$
  4.3 $$\begin{aligned}&\langle [S^\epsilon _\eta , S^\epsilon _\xi ]X, Y\rangle = R^\perp (X,Y,\eta ,\xi ),\end{aligned}$$
  4.4 $$\begin{aligned}&\bar{\nabla }_Y B^\epsilon (X,Z)-\bar{\nabla }_X B^\epsilon (Y,Z) = 0 \end{aligned}$$
  in the distributional sense, where $S^\epsilon$ is the shape operator corresponding to $B^\epsilon$ (Note that $\nabla ^{\perp ,\epsilon }$ is implicit in the above equations).

Then, after passing to a subsequence, $\{(B^\epsilon ,\nabla ^{\perp ,\epsilon })\}$ converges weakly in $L^p$ to a pair $(B,\nabla ^\perp )$ that is still a weak solution of the GCR equations (2.15)–(2.17).

This result can be regarded as a global version on Riemannian manifolds of Theorem 3.3 in Chen–Slemrod–Wang [8]. In this paper, both the statement and the proof for this weak rigidity theorem are global, intrinsic, independent of the local coordinates of the Riemannian manifolds, which offers further geometric insights into the GCR equations.

#### Remark 4.1

Theorem 4.1 for the exact solutions can be extended to the weak rigidity of approximate solutions $(B^\epsilon ,\nabla ^{\perp ,\epsilon })$ of the GCR equations. More precisely, instead of (4.2)–(4.4) in (ii), let $(B^\epsilon ,\nabla ^{\perp ,\epsilon })$ solve the following approximate GCR equations in the distributional sense: For any $X,Y,Z,W \in \Gamma (TM)$ and $\eta ,\xi \in \Gamma (TM^\perp )$,

4.5 $$\begin{aligned}&\langle B^\epsilon (X,W), B^\epsilon (Y,Z)\rangle -\langle B^\epsilon (Y,W), B^\epsilon (X,Z) \rangle +R(X,Y,Z,W) =O_1^\epsilon , \nonumber \\ \end{aligned}$$

4.6 $$\begin{aligned}&\langle [S^\epsilon _\eta , S^\epsilon _\xi ]X, Y\rangle - R^\perp (X,Y,\eta ,\xi )=O_2^\epsilon , \end{aligned}$$

4.7 $$\begin{aligned}&\bar{\nabla }_Y B^\epsilon (X,Z) - \bar{\nabla }_X B^\epsilon (Y,Z) = O_3^\epsilon , \end{aligned}$$

such that $\lim _{\epsilon \rightarrow 0} O_i^\epsilon =0$ in $W^{-1, r}_\mathrm{loc}$ for some $r>1$. Then, after passing to a subsequence, $\{(B^\epsilon ,\nabla ^{\perp ,\epsilon })\}$ converges weakly in $L^p$ to a pair $(B,\nabla ^\perp )$ that is a weak solution of the GCR equations (2.15)–(2.17).

### First Formulation: Identification of the Tensor Fields with Special Div-Curl Structure on Riemannian Manifolds

Now we begin the proof of Theorem 4.1. First we seek tensor fields with special *div-curl structure* for the GCR equations on manifolds.

Recall our previous convention: $X,Y,Z,\ldots \in \Gamma (TM)$ and $\xi ,\eta ,\beta ,\ldots \in \Gamma (TM^\perp )$. For each fixed $(Z,\eta ,\xi )$, define the tensor fields $V^{(B)}_{Z,\eta }, V^{(\nabla ^\perp )}_{\xi ,\eta }: \Gamma (TM)\times \Gamma (TM)\mapsto \Gamma (TM)$ and 1-forms $\Omega ^{(B)}_{Z,\eta }, \Omega ^{(\nabla ^\perp )}_{\xi ,\eta } \in \Omega ^1(M)=\Gamma (T^*M)$ by

4.8 $$\begin{aligned}&V^{(B)}_{Z,\eta }(X,Y):= B(X,Z,\eta )Y-B(Y,Z,\eta )X, \end{aligned}$$

4.9 $$\begin{aligned}&V^{(\nabla ^\perp )}_{\xi ,\eta }(X,Y):=\langle \nabla ^\perp _Y \xi , \eta \rangle X - \langle \nabla ^\perp _X \xi , \eta \rangle Y, \end{aligned}$$

4.10
4.11To avoid further notations, we denote the vector fields canonically isomorphic to $\Omega ^{(B)}_{Z,\eta }$ and $\Omega ^{(\nabla ^\perp )}_{\xi ,\eta }$ (via $\sharp$ and $\flat$) by the same symbols.

Our geometric picture is as follows: The *V*-tensors take two tangential vector fields (*X*, *Y*) to a vector field spanned by *X* and *Y*, which are anti-symmetric in (*X*, *Y*). Thus, $V^{(B)}_{Z,\eta }(X,Y)$ and $V^{(\nabla ^\perp )}_{\xi ,\eta }(X,Y)$ are precisely the *rate of rotations* of $\Omega ^{(B)}_{Z,\eta }$ and $\Omega ^{(\nabla ^\perp )}_{\xi ,\eta }$ in the 2-planes generated by (*X*, *Y*).

The 1-forms $(\Omega ^{(B)},\Omega ^{(\nabla ^\perp )})$ are simply contractions of $(B,\nabla ^\perp )$, and the tensors $(V^{(B)}, V^{(\nabla ^\perp )})$ can be obtained by applying the $\Omega$-tensors to the 2-Grassmannian of *TM*.

Our first formulation concerns the *divergence* of *V* in Eqs. (4.8)–(4.9) and the *curl* (as 2-forms) of $\Omega$ in Eqs. (4.10)–(4.11):

#### Lemma 4.1

(First Formulation) The divergence of *V* and the curl of $\Omega$ can be reformulated as

4.12 $$\begin{aligned}&\mathrm{div}\big (V^{(B)}_{Z,\eta }(X,Y)\big ) = Y B(X,Z,\eta ) - X B(Y,Z,\eta )+B(X,Z,\eta )\mathrm{div}Y\nonumber \\&\qquad \quad -B(Y,Z,\eta )\mathrm{div X},\qquad \quad \end{aligned}$$

4.13 $$\begin{aligned}&\mathrm{div}\big (V^{(\nabla ^\perp )}_{\xi ,\eta }(X,Y)\big ) \!=\!-\!Y\langle \nabla ^\perp _X\xi ,\eta \rangle \!+\!X\langle \nabla ^\perp _Y\xi ,\eta \rangle \!+\!\langle \nabla ^\perp _Y\xi ,\eta \rangle \mathrm{div}X\!-\!\langle \nabla ^\perp _X\xi ,\eta \rangle \mathrm{div}Y, \nonumber \\ \end{aligned}$$

4.14 $$\begin{aligned}&d\big (\Omega ^{(B)}_{Z,\eta }\big )(X,Y) = YB(X,Z,\eta ) - XB(Y,Z,\eta ) + B([X,Y],Z,\eta ),\end{aligned}$$

4.15 $$\begin{aligned}&d \big ( \Omega ^{(\nabla ^\perp )}_{\xi ,\eta }\big )(X,Y) = -Y\langle \nabla ^\perp _X\xi ,\eta \rangle +X\langle \nabla ^\perp _Y\xi ,\eta \rangle - \langle \nabla ^\perp _{[X,Y]}\xi ,\eta \rangle . \end{aligned}$$

#### Proof

To show the first two identities, we use Eq. (3.18) to express the divergence in terms of the Lie derivative, which is further computed from Cartan’s formula:

4.16 $$\begin{aligned} \mathcal {L}_X=d\circ \iota _X + \iota _X \circ d, \end{aligned}$$

where $\iota$ is the interior multiplication. Indeed, in local coordinates, we write $X=X^i\partial _i$, $Y=Y^j\partial _j$, and $dV_g = dx^1 \wedge \ldots \wedge dx^n$. Then

$$\begin{aligned} {\left\{ \begin{array}{ll} \iota _X \, dV_g = (-1)^i X^i dx^1\wedge \ldots \wedge \widehat{dx^i}\wedge \ldots \wedge dx^n,\\ \iota _Y \, dV_g = (-1)^j Y^j dx^1\wedge \ldots \wedge \widehat{dx^j}\wedge \ldots \wedge dx^n, \end{array}\right. } \end{aligned}$$

where $\widehat{\cdots }$ denotes the omission of the corresponding term. As a result,

4.17 $$\begin{aligned} *\big (d ( \iota _X \, dV_g)\big ) = \mathrm{div}\, X,\qquad \,\, *\big (d ( \iota _Y \, dV_g)\big ) = \mathrm{div}\,Y, \end{aligned}$$

as $\partial _i X^i = \mathrm{div}\, X$ and $*dV_g = 1$. On the other hand, for any $f : M\rightarrow \mathbb {R}$, we have

4.18 $$\begin{aligned} {\left\{ \begin{array}{ll} *\big (df \wedge (\iota _Y \, dV_g)\big ) = *(\partial _j f Y^j dV_g) = Yf,\\ *\big (df \wedge (\iota _X \, dV_g)\big ) = *(\partial _i f X^i dV_g) = Xf, \end{array}\right. } \end{aligned}$$

where the vector fields *X* and *Y* are identified with the directional derivatives. Therefore, we conclude

$$\begin{aligned} \mathrm{div}\, V^{(B)}_{Z,\eta }(X,Y)&= *\big (\mathcal {L}_{V^{(B)}_{Z,\eta }(X,Y)} dV_g\big )\\&= *\big (d \iota _{V^{(B)}_{Z,\eta }(X,Y)} \, dV_g \big ) \\&= *\big \{d\big (B(X,Z,\eta )\iota _Y\, dV_g - B(Y,Z,\eta )\iota _X \,dV_g\big ) \big \}\\&= *\big \{d\big (B(X,Z,\eta )\wedge (\iota _Y dV_g)\big )\big \} + B(X,Z,\eta ) *\big (d (\iota _Y \, dV_g)\big ) \\&\qquad - *\big \{d\big (B(Y,Z,\eta )\wedge (\iota _X dV_g)\big )\big \} - B(Y,Z,\eta ) *\big (d (\iota _X \, dV_g)\big )\\&= Y B(X,Z,\eta ) - X B(Y,Z,\eta )+B(X,Z,\eta )\mathrm{div}\,Y-B(Y,Z,\eta )\mathrm{div}\, X. \end{aligned}$$

We use Cartan’s formula (4.16) in the first line, $d(dV_g)=0$ in the second, the definition of $V^{(B)}$ in the third, the definition of *d* in the fourth, and we apply Eqs. (4.17)–(4.18) in the last line, so that Eq. (4.12) is established. Moreover, the proof of Eq. (4.13) is analogous.

For the *generalised curl* (*i.e.*, *d*), recall the identity from §2.2.1:

$$\begin{aligned} d\alpha (X,Y):= X\alpha (Y)-Y\alpha (X) - \alpha ([X,Y]) \qquad \text{ for } \alpha \in \Omega ^1(M). \end{aligned}$$

Applying the above to $\alpha =\Omega ^{(B)}_{Z,\eta }$, we have

$$\begin{aligned} d\Omega ^{(B)}_{Z,\eta }(X,Y)&= X \Omega ^{(B)}_{Z,\eta }(Y) - Y\Omega ^{(B)}_{Z,\eta }(X) - \Omega ^{(B)}_{Z,\eta }([X,Y])\\&= -X B(Y,Z,\eta ) + YB(X,Z,\eta ) + B([X,Y],Z,\eta ), \end{aligned}$$

which is Eq.  (4.14). The proof of Eq. (4.15) is analogous. This completes the proof.

As a remark, it is crucial to recognize that $\delta \big (V^{(B)}_{Z,\eta }(X,Y)\big )$ and $d\big (\Omega ^{(B)}_{Z,\eta }\big )(X,Y)$, as well as $\delta \big (V^{(\nabla ^\perp )}_{\xi ,\eta }(X,Y)\big )$ and $d\big ( \Omega ^{(\nabla ^\perp )}_{\xi ,\eta }\big )(X,Y)$, are essentially the same. They only differ by a zero-th order term involving the Lie bracket [*X*, *Y*]. This observation turns out to be crucial in the proof of Theorem 4.1.

### Second Formulation: The Div-Curl Structure of the GCR Equations

We now express the GCR equations in another form, which is suitable for applying the intrinsic div-curl lemma, i.e., Theorem 3.3. To achieve this, we employ the geometric quantities $V^{(B)}, \Omega ^{(B)},V^{(\nabla ^\perp )}$, and $\Omega ^{(\nabla ^\perp )}$, in the reduced GCR equations (2.18)–(2.20) in Theorem 2.2 to obtain

#### Lemma 4.2

(Second Formulation) The Gauss, Codazzi, and Ricci equations are equivalent to the following equations, respectively:

4.19 $$\begin{aligned}&\sum _\eta \langle V^{(B)}_{Z,\eta }(X,Y), \Omega ^{(B)}_{W,\eta } \rangle = R(X,Y,Z,W), \end{aligned}$$

4.20 $$\begin{aligned}&d(\Omega ^{(B)}_{Z,\eta })(X,Y) + \sum _\beta \langle V^{(\nabla ^\perp )}_{\eta ,\beta }(X,Y),\Omega ^{(B)}_{Z,\beta }\rangle + E(B)=0, \end{aligned}$$

4.21 $$\begin{aligned}&d(\Omega ^{(\nabla ^\perp )}_{\xi ,\eta })(X,Y) + \sum _{\beta } \langle V^{(\nabla ^\perp )}_{\eta ,\beta }(X,Y), \Omega ^{(\nabla ^\perp )}_{\xi ,\beta } \rangle = \sum _Z\langle V^{(B)}_{Z,\xi }(X,Y),\Omega ^{(B)}_{Z,\eta }\rangle , \nonumber \\ \end{aligned}$$

where $E(B):=B(Y,\nabla _XZ,\eta )-B(X,\nabla _YZ,\eta )$ is linear in *B*, and all the summations are at most countable and locally finite.

#### Proof

We divide the proof into four steps.

**1.** We start with Eq. (4.19). Now consider the following spanning set of normal vector fields:

4.22 $$\begin{aligned} \mathcal {S}_1:=\big \{\{\eta _j\}_{j=1}^k\subset \Gamma (TM^\perp ): |\eta _j|=1, \text {span}\{\eta _j\}_{j=1}^k = \Gamma (TM^\perp )\big \}, \end{aligned}$$

where $n+k$ is the dimension of the target space of the isometric immersion.

Since metric *g* is in $W^{1,p}$ on *M*, and the second fundamental form and the normal connection are well defined in $L^p$, then $\mathcal {S}_1$ exists *a.e.* on *M*. In view of the remark above, we can make the following computation in the distributional sense:

4.23 $$\begin{aligned}&\langle B(X,W), B(Y,Z)\rangle -\langle B(Y,W), B(X,Z) \rangle \nonumber \\&\quad = \sum _{\eta \in \mathcal {S}_1}\big (B(X,W,\eta ) B(Y,Z,\eta ) - B(Y,W,\eta ) B(X,Z,\eta )\big ) \nonumber \\&\quad =\sum _{\eta \in \mathcal {S}_1} B\big (B(X,W,\eta )Y - B(Y,W,\eta )X,Z,\eta \big )\nonumber \\&\quad = \sum _{\eta \in \mathcal {S}_1} \Big \langle V^{(B)}_{W,\eta }(X,Y), \Omega ^{(B)}_{Z,\eta } \Big \rangle , \end{aligned}$$

where the last equality follows from the definition of $V^{(B)}$ and $\Omega ^{(B)}$. Combining the above computation with (2.18), we conclude Eq. (4.19).

**2.** Next, we establish Eq. (4.20). Let us start from the Codazzi equation (2.19) in the form of Theorem 2.2:

$$\begin{aligned} XB (Y,Z,\eta ) - YB(X,Z,\eta )&= B([X,Y],Z,\eta ) - B(X,\nabla _YZ,\eta )\\&\quad - B(X,Z,\nabla ^\perp _Y\eta ) + B(Y,\nabla _XZ,\eta ) + B(Y,Z,\nabla ^\perp _X\eta ). \end{aligned}$$

By Eq. (4.10) in the first formulation, we can transform the above equation to the following:

4.24 $$\begin{aligned} d\big (\Omega ^{(B)}_{Z,\eta }\big )(X,Y) \!-\! B(X,\nabla _YZ,\eta ) \!-\! B(X,Z,\nabla ^\perp _Y\eta ) \!+\! B(Y,\nabla _XZ,\eta ) + B(Y,Z,\nabla ^\perp _X\eta )=0. \end{aligned}$$

Moreover, observe that

4.25 $$\begin{aligned} B(Y,Z,\nabla ^\perp _X\eta ) \!- \!B(X,Z,\nabla ^\perp _Y\eta )&= \!\sum _{\beta \in \mathcal {S}_1} \big (B(Y,Z,\beta )\langle \nabla ^\perp _X\eta ,\beta \rangle \!-\! B(X,Z,\beta )\langle \nabla ^\perp _Y\eta ,\beta \rangle \big ) \nonumber \\&=\sum _{\beta \in \mathcal {S}_1} B(\langle \nabla ^\perp _X\eta ,\beta \rangle Y-\langle \nabla ^\perp _Y \eta ,\beta \rangle X, Z,\beta )\nonumber \\&=: \sum _{\beta \in \mathcal {S}_1} \langle V^{(\nabla ^\perp )}_{\eta ,\beta }(X,Y), \Omega ^{(B)}_{Z,\beta }\rangle . \end{aligned}$$

Thus, putting Eqs. (4.24)–(4.25) together, we arrive at Eq. (4.20). It expresses the Codazzi equation in terms of *V* and $\Omega$.

**3**. Finally, we prove Eq. (4.21). The Ricci equation (2.20) in Theorem 2.2 reads

$$\begin{aligned}&X\langle \nabla ^\perp _Y \xi ,\eta \rangle - Y \langle \nabla ^\perp _X\xi ,\eta \rangle \\&\quad = \langle \nabla ^\perp _{[X,Y]}\xi ,\eta \rangle - \langle \nabla ^\perp _X \xi , \nabla ^\perp _Y \eta \rangle + \langle \nabla ^\perp _Y\xi , \nabla ^\perp _X \eta \rangle \\&\qquad \,+ B (\bar{\nabla }_X \xi -\nabla ^\perp _X\xi , Y, \eta ) - B (\bar{\nabla }_X\eta -\nabla ^\perp _X\eta , Y, \xi ). \end{aligned}$$

In light of Eq. (4.15) in the first formulation, we have

4.26 $$\begin{aligned}&d(\Omega ^{(B)}_{\xi ,\eta })(X,Y) + \langle \nabla ^\perp _X \xi , \nabla ^\perp _Y \eta \rangle - \langle \nabla ^\perp _Y\xi , \nabla ^\perp _X \eta \rangle \nonumber \\&\quad = B (\bar{\nabla }_X \xi -\nabla ^\perp _X\xi , Y, \eta ) - B (\bar{\nabla }_Y\eta -\nabla ^\perp _Y\eta , X, \xi ). \end{aligned}$$

The last two terms on the left-hand side can be expressed as

4.27 $$\begin{aligned} \langle \nabla ^\perp _X \xi , \nabla ^\perp _Y \eta \rangle - \langle \nabla ^\perp _Y\xi , \nabla ^\perp _X \eta \rangle&= \sum _{\beta \in \mathcal {S}_1} \big (\langle \nabla ^\perp _X \xi ,\beta \rangle \langle \beta , \nabla ^\perp _Y\eta \rangle - \langle \nabla ^\perp _Y\xi , \beta \rangle \langle \beta , \nabla ^\perp _X\eta \rangle \big ) \nonumber \\&= \sum _{\beta \in \mathcal {S}_1} \langle \nabla ^\perp _{\langle \nabla ^\perp _Y\eta ,\beta \rangle X - \langle \nabla ^\perp _X\eta ,\beta \rangle Y} \xi ,\beta \rangle \nonumber \\&=: \sum _{\beta \in \mathcal {S}_1} \langle V^{(\nabla ^\perp )}_{\eta ,\beta } (X,Y), \Omega ^{(\nabla ^\perp )}_{\xi ,\beta } \rangle , \end{aligned}$$

by using the definition of $V^{(\nabla ^\perp )}$ and $\Omega ^{(\nabla ^\perp )}$ in Eqs. (4.9) and (4.11).

To deal with the right-hand side of Eq. (4.26), we temporarily assume the existence of the tangential spanning set of unit vector fields:

4.28 $$\begin{aligned} \mathcal {S}_2:= \big \{\{Z_j\}_{j=1}^n\subset \Gamma (TM)\,:\, |Z_j|\equiv 1, \text { span}\{Z_j\}_{j=1}^n=\Gamma (TM)\big \}. \end{aligned}$$

In this case, one can compute the right-hand side of equation (4.26):

4.29 $$\begin{aligned}&B (\bar{\nabla }_X \xi - \nabla ^\perp _X\xi , Y, \eta ) - B (\bar{\nabla }_X\eta -\nabla ^\perp _X\eta , Y, \xi )\nonumber \\&\quad = \sum _{Z\in \mathcal {S}_2} \big (B(\langle \bar{\nabla }_X\xi -\nabla ^\perp _X\xi , Z \rangle Z, Y,\eta ) - B(\langle \bar{\nabla }_X\eta -\nabla ^\perp _X\eta , Z \rangle Z, Y,\xi )\big )\nonumber \\&\quad = \sum _{Z\in \mathcal {S}_2} \big (B(Z,X,\eta )B(Z,Y,\xi ) - B(Z,X,\xi )B(Z,Y,\eta )\big ). \end{aligned}$$

Indeed, to obtain the last equality, recall that *B* is symmetric in the first two arguments. Then, using Eqs. (2.10)–(2.11), we have

$$\begin{aligned} \langle \bar{\nabla }_X\xi -\nabla ^\perp _X\xi , Z \rangle = -\langle S_\xi X,Z\rangle = -B(X,Z,\xi ), \end{aligned}$$

where *S* is the shape operator. The other term follows similarly.

On the other hand, by Eqs. (4.8) and (4.10), we have

4.30 $$\begin{aligned}&B(Z,X,\eta )B(Z,Y,\xi ) - B(Z,X,\xi )B(Z,Y,\eta )\nonumber \\&\quad =-B(B(X,Z,\xi )Y - B(Y,Z,\xi )X, Z, \xi )=: \langle V^{(B)}_{Z,\xi }(X,Y), \Omega ^{(B)}_{Z,\eta } \rangle . \end{aligned}$$

Thus, putting (4.26)–(4.27) and (4.29)–(4.30) together, we obtain Eq. (4.21), provided that the spanning set $\mathcal {S}_2 \subset \Gamma (TM)$ is well defined.

**4.** Unfortunately, we cannot take the existence of $\mathcal {S}_2$ for granted. For example, on $M=\mathbb {S}^{2m}$, the Hairy Ball theorem shows that any $Z\in \Gamma (TM)$ must vanish at some point. To overcome this difficulty, we may resort to a partition of unity argument. Let $\mathcal {A}=\{U_\alpha :\alpha \in \mathcal {I}\}$ be an atlas for *M*, and let $\{\rho _\alpha :\alpha \in \mathcal {I}\}$ be a smooth partition of unity subordinate to $\mathcal {A}$ (*cf.* Sect. 2). On each $U_\alpha$, the spanning set in the form of $\mathcal {S}_2$ exists, since $U_\alpha$ is diffeomorphic to $\mathbb {R}^n$. We write

$$\begin{aligned} \mathcal {S}_2^\alpha =\big \{\{Z^\alpha _j\}_{j=1}^n\subset \Gamma (TU_\alpha )\; :\; |Z^\alpha _j|\equiv 1, \text {span}\{Z^\alpha _j\}_{j=1}^n=\Gamma (TU_\alpha ) \big \}. \end{aligned}$$

Define

4.31 $$\begin{aligned} \widetilde{\mathcal {S}_2}= \big \{Z:=\sum _{\alpha \in \mathcal {I}} \rho _\alpha Z^\alpha \chi _{\text {supp}(\rho _\alpha )}\; :\; Z^\alpha \in \mathcal {S}_2^\alpha , \alpha \in \mathcal {I}\big \}. \end{aligned}$$

Then we can take $\widetilde{\mathcal {S}_2}$ in place of $\mathcal {S}_2$ so that all the preceding arguments for the Ricci equation pass through. This completes the proof. $\square$

### Proof of Theorem 4.1

We can now prove the global weak rigidity theorem, Theorem 4.1, for the GCR equations on Riemannian manifolds. In fact, the main ingredients of the proof have been provided in the previous two formulations, which express the GCR equations in the form suitable for employing Theorem 3.3.

#### Proof of Theorem 4.1

The proof consists of three steps.

**1.** By taking the orientable double cover when necessary, we may assume that *M* is orientable. Then we can employ Theorem 3.3 on *M*. Moreover, by Theorems 2.1–2.2, $(B^\epsilon ,\nabla ^{\perp ,\epsilon })$ are solutions of Eqs. (2.18)–(2.20) in the distributional sense.

**2.** Consider our second formulation. The zero-th order terms of Eqs. (4.19)–(4.21) contain linear combinations of the following quadratic nonlinear forms:

4.32 $$\begin{aligned} \begin{aligned}&\langle V^{(B^\epsilon )}_{W,\eta }(X,Y), \Omega ^{(B^\epsilon )}_{Z,\eta }\rangle , \quad \langle V^{(\nabla ^{\perp ,\epsilon })}_{\eta ,\beta }(X,Y),\Omega ^{(\nabla ^{\perp ,\epsilon })}_{\xi ,\beta }\rangle ,\\&\langle V^{(B^\epsilon )}_{Z,\xi }(X,Y),\Omega ^{(B^\epsilon )}_{Z,\eta } \rangle , \quad \langle V^{(\nabla ^{\perp ,\epsilon })}_{\eta ,\beta }(X,Y),\Omega ^{(B^\epsilon )}_{Z,\beta }\rangle , \end{aligned} \end{aligned}$$

and the linear term $E(B^\epsilon )$ in $B^\epsilon$. Clearly, $E(B^\epsilon )\rightarrow E(B)$ in $\mathcal {D}'$, owing to the linearity of *B* and the uniform $L^p$ boundedness of $\{B^\epsilon \}$.

For the four terms in (4.32), by the hypotheses of Theorem 4.1, we find that

$$\begin{aligned} \{V^{(B^\epsilon )}(X,Y), \Omega ^{(B^\epsilon )}, V^{(\nabla ^{\perp ,\epsilon })}(X,Y), \Omega ^{(\nabla ^{\perp ,\epsilon })}\} \end{aligned}$$

are uniformly bounded in $L^p_\mathrm{loc}$. As a consequence, the four quadratic terms in (4.32) are uniformly bounded in $L^{p/2}_\mathrm{loc}$ for $p>2$.

Then, by Eqs. (4.20)–(4.21), we know that $d(\Omega ^{(\nabla ^{\perp ,\epsilon })}_{\xi ,\eta })(X,Y)$ and $d(\Omega ^{(B^\epsilon )}_{Z,\eta })(X,Y)$ are uniformly bounded in $L^{p/2}_\mathrm{loc}$, so that they are compact at least in $W^{-1, p'}_\mathrm{loc}$ for some $p'\in (1, 2)$ by the Sobolev embedding. On the other hand, since $\Omega ^{(\nabla ^{\perp ,\epsilon })}_{\xi ,\eta }$ and $\Omega ^{(B^\epsilon )}_{Z,\eta }$ are uniformly bounded in $L^p_\mathrm{loc}$, $d(\Omega ^{(\nabla ^{\perp ,\epsilon })}_{\xi ,\eta })(X,Y)$ and $d(\Omega ^{(B^\epsilon )}_{Z,\eta })(X,Y)$ are uniformly bounded in $W^{-1,p}_\mathrm{loc}, p>2$. Then, by interpolation, we conclude that

$$\begin{aligned} \big \{d(\Omega ^{(\nabla ^{\perp ,\epsilon })}_{\xi ,\eta })(X,Y), \,\, d(\Omega ^{(B^\epsilon )}_{Z,\eta })(X,Y)\big \} \qquad \,\,\, \hbox {are pre-compact subsets of } H^{-1}_\mathrm{loc}(M). \end{aligned}$$

Furthermore, Eqs. (4.12)–(4.15) of the first formulation lead to the following identities:

$$\begin{aligned} \mathrm{div}\big (V^{(B^\epsilon )}_{Z,\eta }(X,Y)\big )= & {} d(\Omega ^{(\nabla ^{\perp ,\epsilon })}_{Z,\eta })(X,Y)-B^\epsilon ([X,Y],Z,\eta )\nonumber \\&+B^\epsilon (X,Z,\eta )\mathrm{div}Y-B^\epsilon (Y,Z,\eta )\mathrm{div}X,\qquad \quad \\ \mathrm{div}\big (V^{(\nabla ^{\perp ,\epsilon })}_{\xi ,\eta }(X,Y)\big )= & {} d(\Omega ^{(\nabla ^{\perp ,\epsilon })}_{\xi ,\eta })(X,Y) + \langle \nabla ^{\perp ,\epsilon }_{[X,Y]}\xi ,\eta \rangle +\langle \nabla ^{\perp ,\epsilon }_Y\xi ,\eta \rangle \mathrm{div}X\nonumber \\&-\,\langle \nabla ^{\perp ,\epsilon }_X\xi , \eta \rangle \mathrm{div}Y. \end{aligned}$$

Since $(B^\epsilon ,\nabla ^{\perp ,\epsilon })$ are uniformly bounded in $L^p_\mathrm{loc}, p>2$, again by the Sobolev embeddings, they are compact in $H^{-1}_\mathrm{loc}$. Since the divergence operator equals $\delta$ (the adjoint of *d*) modulo a sign, we conclude that

$$\begin{aligned} \big \{\delta \big (V^{(B^\epsilon )}_{Z,\eta }(X,Y)\big ), \,\,\delta \big (V^{(\nabla ^{\perp ,\epsilon })}_{\xi ,\eta }(X,Y)\big )\big \} \quad \,\, \hbox {are pre-compact sets of }H^{-1}_\mathrm{loc}(M). \end{aligned}$$

**3.** Since $(B^\epsilon ,\nabla ^{\perp ,\epsilon })$ are uniformly bounded in $L^p_\mathrm{loc}, p>2$, after passing to subsequences, they converge to some $(B,\nabla ^\perp )$ weakly in $L^p_\mathrm{loc}$. Combining the arguments in Step 2 with Theorem 3.3, we obtain the following convergence in the distributional sense:

$$\begin{aligned}&\langle V^{(B^\epsilon )}_{W,\eta }(X,Y), \Omega ^{(B^\epsilon )}_{Z,\eta }\rangle \longrightarrow \langle V^{(B)}_{W,\eta }(X,Y), \Omega ^{(B)}_{Z,\eta }\rangle ,\\&\langle V^{(\nabla ^{\perp ,\epsilon })}_{\eta ,\beta }(X,Y),\Omega ^{(\nabla ^{\perp ,\epsilon })}_{\xi ,\beta }\rangle \longrightarrow \langle V^{(\nabla ^\perp )}_{\eta ,\beta }(X,Y),\Omega ^{(\nabla ^\perp )}_{\xi ,\beta }\rangle ,\\&\langle V^{(B^\epsilon )}_{Z,\xi }(X,Y),\Omega ^{(B^\epsilon )}_{Z,\eta } \rangle \longrightarrow \langle V^{(B)}_{Z,\xi }(X,Y),\Omega ^{(B)}_{Z,\eta } \rangle ,\\&\langle V^{(\nabla ^{\perp ,\epsilon })}_{\eta ,\beta }(X,Y),\Omega ^{(B^\epsilon )}_{Z,\beta }\rangle \longrightarrow \langle V^{(\nabla ^\perp )}_{\eta ,\beta }(X,Y),\Omega ^{(B)}_{Z,\beta }\rangle . \end{aligned}$$

Therefore, in Eqs. (4.19)–(4.21) in the second formulation, we can pass the limits as $\epsilon \rightarrow 0$. This guarantees that the weak limit $(B,\nabla ^\perp )$ is still a weak solution of the GCR system. This completes the proof. $\square$

To conclude this section, we remark that Eqs. (2.1)–(2.3) in [8], which are the Gauss, Codazzi, and Ricci equations in local coordinates, can be seen directly from our global formulations in Theorems 2.1–2.2.

Indeed, write $\{\partial _i:1\le i \le n\}$ as a local coordinate system on the tangent bundle *TM*, and $\{\partial _\alpha : {n+1} \le \alpha \le {n+k}\}$ for a local coordinate system on the normal bundle $TM^\perp$. To write the GCR equations in the local coordinates, we define

4.33 $$\begin{aligned} h_{ij}^\alpha := \langle B(\partial _i, \partial _j), \partial _\alpha \rangle , \qquad \kappa _{i\beta }^\alpha := \langle \nabla ^\perp _{\partial _\alpha } \partial _i, \partial _\beta \rangle , \end{aligned}$$

and substitute the unit vector fields $\partial _i$ (or $\partial _\alpha$) in place of *X* (or $\xi$, respectively), in the GCR equations in Theorems 2.1–2.2. We solve for $\{h_{ij}^\alpha ,\kappa _{i\beta }^\alpha \}$, namely the second fundamental form and the normal affine connection written coordinate-wise. In this way, we recover Eqs. (2.1)–(2.3) in [8]:

4.34 $$\begin{aligned}&h^\alpha _{ik} h^\alpha _{jl} - h^\alpha _{il} h^\alpha _{jk} = R_{ijkl}, \end{aligned}$$

4.35 $$\begin{aligned}&\frac{\partial h^\alpha _{lj}}{\partial x^k} - \frac{\partial h^\alpha _{kj}}{\partial x^l} + \Gamma ^m_{lj}h^\alpha _{km} - \Gamma ^m_{kj}h^\alpha _{lm} + \kappa ^\alpha _{k\beta }h^\beta _{lj} - \kappa ^\alpha _{l\beta }h^\beta _{kj} = 0, \end{aligned}$$

4.36 $$\begin{aligned}&\frac{\partial \kappa ^\alpha _{l\beta }}{\partial x^k} - \frac{\partial \kappa ^\alpha _{k\beta }}{\partial x^l} - g^{mn}\big (h^\alpha _{ml}h^\beta _{kn}-h^\alpha _{mk}h^\beta _{ln}\big ) + \kappa ^\alpha _{k\gamma }\kappa ^\gamma _{l\beta } -\kappa ^\alpha _{l\gamma }\kappa ^\gamma _{k\beta }=0, \end{aligned}$$

where $R_{ijkl}:=R(\partial _i,\partial _j,\partial _k,\partial _l)$, and the Christoffel symbols are defined as usual by

$$\begin{aligned} \Gamma ^k_{ij}:=\frac{1}{2}g^{kl} \big (\partial _i g_{jl} + \partial _j g_{il} - \partial _l g_{ij}\big ). \end{aligned}$$

Therefore, Theorem 4.1 for the weak rigidity of the GCR equations is a global, intrinsic version of the local weak rigidity result of Theorem 3.3 in [8].

## Isometric Immersions, the Cartan Formalism, and the GCR Equations on Manifolds with Lower Regularities

In Sect. 4, we have proved the global weak rigidity of the GCR equations on Riemannian manifolds with lower regularity. Then the next natural question is about its geometric implications. We address this question with two interrelated goals:

(i) Global isometric immersions for simply connected manifolds with lower regularity are constructed from the GCR equations. As remarked in the introduction, this is related to the *realization problem* in elasticity.
(ii) The global weak rigidity of isometric immersions in turn provides crucial insights to the global weak rigidity of the GCR equations. This observation will lead to an alternative proof of Theorem 4.1; *cf.* Sect. 7.1.

### From PDEs to Geometry: An Equivalence Theorem

We now address the central question raised above. First, it should be noticed that the results in Sect. 4 are essentially *PDE-theoretic*. Despite the geometric—global and intrinsic—nature of the formulation and proof of Theorem 4.1, we have only analyzed the GCR equations *per se*, but have not referred to their geometric origin, i.e., the isometric immersion problem. One would expect that, providing that our formulation is natural, the weak rigidity of isometric immersions and the weak rigidity of the GCR equations should be essentially the same problem. We now formalize this observation and prove it in mathematical rigor.

We point out that the *realization problem*, i.e., the construction of isometric immersions from the GCR equations, has been investigated in the recent years; see Ciarlet–Gratie–Mardare [9], Mardare [30]–[32], Szopos [44], and the references cited therein. These previous results, which solve the *realization problem* locally, can be summarized in the following theorem.

#### Theorem 5.1

Let $U\subset \mathbb {R}^n$ be a simply connected open set. Suppose that the symmetric matrix fields $\{g_{ij}\}\in W^{1,p}_\mathrm{loc}(U;O(n))$ and $\{h_{ij}=(h^{\alpha }_{ij})\}_{n+1\le \alpha \le n+k}\subset L^p_\mathrm{loc}(U;O(n))$, and the anti-symmetric matrix field $\{\kappa _{ij}=(\kappa ^\alpha _{ij})\}_{n+1\le \alpha \le n+k} \subset L^p_\mathrm{loc}(U;\mathfrak {so}(n))$ prescribed on *U* satisfy the GCR equations (4.34)–(4.36) in local coordinates in the distributional sense. Then there exists a $W^{2,p}_\mathrm{loc}$ isometric immersion $f: U\hookrightarrow \mathbb {R}^{n+k}$ such that $g_{ij}, h_{ij}$, and $\kappa _{ij}$ are its metric, second fundamental form, and normal connection in local coordinates, respectively.

Here and in the sequel, we write $\mathfrak {gl}(q;\mathbb {R})$ for the space of $q\times q$ matrices with real entries, $O(q) \subset \mathfrak {gl}(q;\mathbb {R})$ for the space of symmetric $q \times q$ matrices, and $\mathfrak {so}(q)\subset \mathfrak {gl}(q;\mathbb {R})$ the space of $q\times q$ anti-symmetric matrices.

#### Remark 5.1

The codimension *k* of the isometric immersion in Theorem 5.1 above is required to be larger than or equal to the minimal *Janet dimension* $J(n):=\frac{n(n+1)}{2}$. For more details, we refer to Janet [25] and the exposition by Han–Hong [24].

The strategy for the proof in [30]–[32] and [44] can be briefly sketched as follows: First, the GCR equations can be transformed into two types of first-order matrix-valued PDE systems with $W^{2,p}$ coefficients, known as the Pfaff and Poincaré systems; then, applying various analytic results established in [30]–[32], one can construct explicitly the local isometric immersions by solving the Pfaff and Poincaré systems with the rough coefficients. Nevertheless, despite the successful solution to the *realization problem* (at least locally), the transformations from the GCR equations to the Pfaff and Poincaré systems in [30]–[32] and [44] appear quite delicate, which involve many different types of geometric quantities in local coordinates (e.g., metrics, connections, and curvatures) as the entries of the same matrix of enormous size.

In this section, we give an alternative global geometric proof of Theorem 5.1. In addition, we solve the problems listed in goals (i) and (ii) at one stroke. Our perspective is essentially different from those in [30]–[32] and [44]: We aim at establishing the equivalence of the GCR equations and the existence of isometric immersions on Riemannian manifolds with $W^{1,p}_\mathrm{loc}$ metric, $p>n$, via the Cartan formalism for exterior differential calculus (see [43]).

We first state the main theorem of this section, which concerns the equivalence of three formulations of the GCR equations *in disguise*. Roughly speaking, we view the Cartan formalism as the bridge between the GCR equations and isometric immersions.

#### Theorem 5.2

Let (*M*, *g*) be an *n*-dimensional, simply connected Riemannian manifold with metric $g\in W^{1,p}_\mathrm{loc}$, $p>n$, and let $(E,M,\mathbb {R}^k)$ be a vector bundle over *M*. Assume that *E* has a $W^{1,p}_\mathrm{loc}$ metric $g^E$ and an $L^p_\mathrm{loc}$ connection $\nabla ^E$ such that $\nabla ^E$ is compatible with metric $g^E$. Moreover, suppose that there exists an $L^p_\mathrm{loc}$ tensor field $S:\Gamma (E) \times \Gamma (TM)\mapsto \Gamma (TM)$ satisfying

$$\begin{aligned} \langle X, S_\eta (Y)\rangle - \langle S_\eta (X), Y\rangle =0 \end{aligned}$$

with the corresponding $L^p_\mathrm{loc}$ tensor field $B: \Gamma (TM)\times \Gamma (TM)\mapsto \Gamma (E)$ defined by

$$\begin{aligned} \langle B(X,Y),\eta \rangle = -\langle S_\eta (X), Y \rangle . \end{aligned}$$

Then the following are equivalent:

(i) The GCR equations as in Theorem 2.1 with $R^\perp$ replaced by $R^E$, the Riemann curvature operator on the bundle;
(ii) The Cartan formalism (5.1)–(5.5);
(iii) The existence of a global isometric immersion $f\in W^{2,p}_\mathrm{loc}(M; \mathbb {R}^{n+k})$ such that the induced normal bundle $T(fM)^\perp$, normal connection $\nabla ^\perp$, and second fundamental form can be identified with $E, \nabla ^E$, and *B*, respectively.

In (i)–(ii), the equalities are taken in the distributional sense and, in (iii), the isometric immersion $f\in W^{2,p}_\mathrm{loc}$ is unique *a.e.*, modulo the group of Euclidean motions $G=\mathbb {R}^{n+k} \rtimes O(n+k)$.

In Theorem 5.2, we require $p>n$ (instead of $p>2$ for the weak rigidity of the GCR equations, as in Sect. 4) to guarantee that the immersion is $C^1$, which agrees with the classical notions from differential geometry. In Sect. 5.2, the Cartan formalism is introduced. This clarifies the precise meaning for the second item in the above theorem. Then, in Sect. 5.3, we give a proof of Theorem 5.2.

Finally, the implication: $\mathrm{(i)} \Rightarrow \mathrm{(iii)}$ leads to the following global realization theorem on Riemannian manifolds, independent of the local coordinates:

#### Corollary 5.1

Let (*M*, *g*) be an *n*-dimensional, simply connected Riemannian manifold with metric $g\in W^{1,p}_\mathrm{loc}, p>n,$ and let $(E,M,\mathbb {R}^k)$ be a vector bundle over *M*. Assume that *E* has a $W^{1,p}_\mathrm{loc}$ metric $g^E$ and an $L^p_\mathrm{loc}$ connection $\nabla ^E$ such that $\nabla ^E$ is compatible with metric $g^E$. Moreover, suppose that there exists an $L^p_\mathrm{loc}$ tensor field $S:\Gamma (E) \times \Gamma (TM)\mapsto \Gamma (TM)$ satisfying

$$\begin{aligned} \langle X, S_\eta (Y)\rangle - \langle S_\eta (X), Y\rangle =0 \end{aligned}$$

with the corresponding $L^p_\mathrm{loc}$ tensor field $B: \Gamma (TM)\times \Gamma (TM)\mapsto \Gamma (E)$ defined by

$$\begin{aligned} \langle B(X,Y),\eta \rangle = -\langle S_\eta (X), Y \rangle . \end{aligned}$$

Assume that the GCR equations (as in Theorem 2.1, with $R^\perp$ replaced by $R^E$) are satisfied in the distributional sense. Then there exists a global isometric immersion $f \in W^{2,p}_\mathrm{loc}(M; \mathbb {R}^{n+k})$ such that $TM^\perp$ is identified with *E* (together with the metric and connection), and *B* and *S* coincide with the second fundamental form and the shape operator associated with *f*.

### Cartan Formalism

We now introduce a useful tool, the Cartan formalism, for isometric immersions. We sketch some results directly pertaining to isometric immersions. For an local isometric immersion $f: U\subset M^n \hookrightarrow \mathbb {R}^{n+k}$, we adopt the index convention as in [47]:

$$\begin{aligned} 1\le i,j \le n;\qquad 1\le a,b,c,d,e \le n+k; \qquad n+1 \le \alpha ,\beta ,\gamma \le n+k. \end{aligned}$$

In the sequel, our setting is always the same as in Theorem 5.2.

On a local chart $U \subset M$ where the vector bundle *E* is trivialized (i.e., $E|_U$ is diffeomorphic to $U\times \mathbb {R}^k$), let $\{\omega ^i\} \subset \Omega ^1(U)$ be an orthonormal co-frame dual to the orthonormal frame $\{\partial _i\} \subset \Gamma (TU)$. The latter is called a moving frame adapted to *U*. The Cartan formalism is thus also known as the *method of moving frames*.

It is well known that the GCR equations are equivalent to the following two systems:

5.1 $$\begin{aligned} d\omega ^i= & {} \sum _j \omega ^j \wedge \omega ^i_j, \end{aligned}$$

5.2 $$\begin{aligned} d\omega ^a_b= & {} - \sum _c \omega _b^c \wedge \omega ^a_c, \end{aligned}$$

where (5.1)–(5.2) are known as the first and second structural equations (*cf.* [10, 43]). The 1-forms $\{\omega ^a_b\}$ are defined as follows: Let $\{\eta _{n+1}, \ldots , \eta _{n+k}\} \subset \Gamma (E)$ be an orthonormal basis for fiber $\mathbb {R}^k$ of bundle *E* over the trivialized chart *U*. Then set

5.3 $$\begin{aligned}&\omega ^i_j (\partial _k):= \langle \nabla _{\partial _k}\partial _j, \partial _i \rangle , \end{aligned}$$

5.4 $$\begin{aligned}&\omega ^i_\alpha (\partial _j)=-\omega ^\alpha _i(\partial _j):=\langle B(\partial _i, \partial _j),\eta _\alpha \rangle , \end{aligned}$$

5.5 $$\begin{aligned}&\omega ^\alpha _\beta (\partial _j):= \langle \nabla ^E_{\partial _j}\eta _\alpha , \eta _\beta \rangle . \end{aligned}$$

The structural equations (5.1)–(5.2), together with the definitions for the connection form in local coordinates, i.e., Eqs. (5.3)–(5.5), are referred to as the Cartan formalism.

It is both convenient and conceptually important to introduce the following short-hand notations: Write

5.6 $$\begin{aligned} W= \{\omega ^a_b\}\in \Omega ^1(U; \mathfrak {so}(n+k)), \end{aligned}$$

which is a field of 1-form-valued anti-symmetric matrices (or equivalently, the field of anti-symmetric matrix-valued 1-forms). Heuristically, *W* packages together the second fundamental form and normal connection. We also write

5.7 $$\begin{aligned} w=(\omega ^1, \ldots , \omega ^n, 0, \ldots , 0)\in \Omega ^1(U; \mathbb {R}^{n+k}). \end{aligned}$$

As a result, the structural equations can be written as

5.8 $$\begin{aligned} dw = w\wedge W, \qquad dW+W\wedge W=0, \end{aligned}$$

where operator $\wedge$ between two matrix-valued differential forms denotes the wedge product of differential forms together with matrix multiplication. In the sequel, we always adhere to the practice of writing (matrix) Lie group-valued PDEs in short-handed forms as in (5.8).

We remark that the structure equations, as well as various other Lie group-valued equations we will introduce below, are *intrinsic*. That is, these equations are independent of the *moving frames*, and hence are natural in coordinate-free notations. This ensures the global and intrinsic nature of the proof of Theorem 5.2, which is the content in Sect. 5.3. For more details on the Cartan formalism, see [43].

### Proof of Theorem 5.2

With the Cartan formalism introduced, we are now in the position to prove the main result of this section, i.e., Theorem 5.2. Our proof is intrinsic and global in nature, i.e., covariant with respect to the change of local frames. We divide the proof in seven steps.

**1.** We first notice the equivalence between the GCR equations and the Cartan formalism (*cf*. [43]). Also, it is well known that the existence of a local isometric immersion implies the GCR equations (*cf*. [13]). Although the above classical results are established in the $C^\infty$ category in [13, 43], it is easy to check that the proofs remain unaltered for the lower regularity case, since $g\in W^{1,p}_\mathrm{loc}$ ensures that all the calculations involved make sense in the distributional sense. Thus, it remains to prove $\mathrm{(ii)}\Rightarrow \mathrm{(iii)}$, i.e., the Cartan formalism implies the existence of an isometric immersion.

We follow closely the arguments in Tenenblat [47] for the $C^\infty$ case. We first prove the local version of the theorem and then extend to the global version on simply connected manifolds via topological arguments.

**2.** Recall that $W=\{\omega ^a_b\}\in \Omega ^1(U; \mathfrak {so}(n+k))$ and $w=(\omega ^1, \ldots , \omega ^n, 0, \ldots , 0)\in \Omega ^1(U; \mathbb {R}^{n+k})$. In order to find an isometric immersion based upon the structural equations (5.1)–(5.2), we first solve for an affine map *A* (taking $\partial _j$ to $\eta _\alpha$), which essentially consists of the components of the normal affine connection, and then the isometric immersion *f* is constructed from *A*.

We first carry out such constructions locally. More precisely, given $P_0\in U$, we find an open set *V* with $P_0 \in V \subset U$ and a field of symmetric matrices $A=\{A^a_b\}: V \mapsto O(n+k)$, satisfying the first-order PDE system:

5.9 $$\begin{aligned} {\left\{ \begin{array}{ll} W = dA \cdot A^\top \qquad \text { in } V,\\ A(P_0)=A_0, \end{array}\right. } \end{aligned}$$

where $A^\top$ is the transpose of matrix *A*. This equation means that

$$\begin{aligned} W(X|_P)=dA(X|_P)\cdot A(P) \qquad \text{ for } P\in V \text{ and } X\in \Gamma (TV), \end{aligned}$$

where the dot is the matrix multiplication.

Now, under the assumption that Eq. (5.9) is satisfied, we next solve for function $f: V \mapsto \mathbb {R}^{n+k}$ such that

5.10 $$\begin{aligned} {\left\{ \begin{array}{ll} df = w \cdot A \qquad \text { in } V,\\ f(P_0)=f_0, \end{array}\right. } \end{aligned}$$

i.e., $df_P(X|_P)=w(X|_P) \cdot A(P)$ for $P\in V$ and $X\in \Gamma (TV)$. Following [31, 32], we call (5.9) the Pfaff system, and call (5.10) the Poincaré system. The names come from the theory of integrable systems and the Poincaré lemma in cohomology theory, respectively.

**3.** In the $C^\infty$ category, to establish the solvability of PDEs in form (5.9)–(5.10), which can be viewed as complete integrability conditions, we only need to check the *involutiveness*, in view of the Frobenius theorem. This constitutes the arguments in [47]. In the lower regularity case, we seek for the correct analogue to the Frobenius theorem. The following lemma can serve for this purpose:

#### Lemma 5.1

(Mardare [32]) The following solubility criteria hold for two types of first-order matrix Lie group-valued PDE systems:

(i) The Pfaff system, Theorem 7 of [31]: Let $U\subset \mathbb {R}^n$ be a simply connected open set, $x_0 \in U$, and $M_0\in \mathfrak {gl}(n; \mathbb {R})$, the space of $n\times n$ real matrices. Then the following system:
  5.11 $$\begin{aligned} \frac{\partial M}{\partial x^i}=Q_i\cdot M, \,\,\, i=1,2,\ldots ,n, \,\,\qquad M(x_0)=M_0, \end{aligned}$$
  with the matrix fields $Q_i\in L^p_\mathrm{loc}(U; \mathfrak {gl}(n;\mathbb {R}))$ for $i=1,2,\ldots , n$, and $p>n$, has a unique solution $M\in W^{1,p}_\mathrm{loc}(U; \mathfrak {gl}(n; \mathbb {R}))$ if and only if the following compatibility condition holds:
  5.12 $$\begin{aligned} \frac{\partial Q_i}{\partial x^j} - \frac{\partial Q_j}{\partial x^i} =[Q_i, Q_j] \qquad \text { in } \mathcal {D}'\,\,\,\text { for each } i,j=1,2, \dots , n. \end{aligned}$$
(ii) The Poincaré system, Theorem 6.5 of [32]: Let $U\subset \mathbb {R}^n$ be a simply connected open set, $x_0 \in U$, and $\psi _0 \in \mathbb {R}$. Then the following system:
  5.13 $$\begin{aligned} \frac{\partial \psi }{\partial x^i} = \phi _i, \,\,\, i=1,2,\ldots , n, \,\qquad \psi (x_0) = \psi _0, \end{aligned}$$
  with $\phi _i \in L^p_\mathrm{loc}(U)$ for $i=1,2,\ldots , n$, and $1 \le p \le \infty$, has a unique solution $\psi \in W^{1,p}_\mathrm{loc}(U)$ if and only if the following compatibility condition holds:
  5.14 $$\begin{aligned} \frac{\partial \phi _i}{\partial x^j} - \frac{\partial \phi _j}{\partial x^i} = 0 \qquad \text { in } \mathcal {D}'\,\,\, \text { for each } i,j=1,2, \dots , n. \end{aligned}$$

We also remark that the uniqueness in (ii) is proved by Schwartz [42]. Thanks to Lemma 5.1, solving for systems (5.9) and (5.10) can be reduced to checking the compatibility conditions in the form of Eqs. (5.12) and (5.14), provided that the regularity assumptions are satisfied.

**4.** Let us first solve for the Pfaff system (5.9). Performing contraction with the moving frame $\{\partial _a\}$, one has

5.15 $$\begin{aligned} dA(\partial _a) = \frac{\partial A}{\partial {x^a}}= W(\partial _a)\cdot A, \end{aligned}$$

which is a Pfaff system on $\mathfrak {gl}(n+k;\mathbb {R})$. By assumption, $g, g^E \in W^{1,p}_\mathrm{loc}$, and $B, \nabla ^E \in L^p_\mathrm{loc}$, so that $W\in L^p_\mathrm{loc}$ with $p>n$. This verifies the regularity hypotheses in Lemma 5.1(i).

The compatibility criterion (5.12) can be written in local coordinates as

5.16 $$\begin{aligned} \partial _b \big (\omega ^c_d(\partial _a)\big ) - \partial _a \big (\omega ^c_d(\partial _b)\big ) = \sum _e [\omega ^c_e (\partial _a), \omega ^e_d(\partial _b)]. \end{aligned}$$

Recall formula (2.1) in Sect. 2: As a special case, for any 1-form $\alpha$ and vector fields (*X*, *Y*),

5.17 $$\begin{aligned} d\alpha (X,Y)= X\alpha (Y)-Y\alpha (X) - \alpha ([X,Y]). \end{aligned}$$

Thus, taking $\alpha =\omega ^c_d$, $X=\partial _b$, and $Y=\partial _a$, we have

5.18 $$\begin{aligned} d\omega ^c_d (\partial _a, \partial _b) = \partial _b \big (\omega ^c_d(\partial _a)\big ) - \partial _a \big (\omega ^c_d(\partial _b)\big ). \end{aligned}$$

Moreover, the Lie bracket on the right-hand side of Eq. (5.16) can be written as the wedge product of 1-forms:

$$\begin{aligned} \sum _e [\omega ^c_e (\partial _a), \omega ^e_d(\partial _b)] = \sum _e \big (\omega ^c_e\wedge \omega ^e_d\big )(\partial _a, \partial _b). \end{aligned}$$

Combining these two observations, the compatibility criterion for the Pfaff system (5.9) is equivalent to

$$\begin{aligned} d\omega ^c_d (\partial _a, \partial _b) = \sum _e \big (\omega ^e_d \wedge \omega ^c_e\big ) (\partial _a, \partial _b). \end{aligned}$$

This is precisely the second structural equation (5.2). Thus, by Lemma 5.1(i), we can find $A\in W^{1,p}_\mathrm{loc}(V; \mathfrak {gl}(n+k;\mathbb {R}))$ satisfying Eq. (5.9). Using equation $W=dA\cdot A^\top$ and the fact that $W\in \mathfrak {so}(n+k)$, we deduce that *A* maps into $O(n+k)$.

**5.** Next, we solve for immersion *f* from the Poincaré system (5.10). Since $w\cdot A \in W^{1,p}_\mathrm{loc}$ with $p>n$, the regularity assumption in Lemma 5.1(ii) is satisfied, both for $w\cdot A$ and its first derivatives. Thus, if we verify the compatibility condition in the form of Eq. (5.14), we can establish the existence of $f\in W^{2,p}_\mathrm{loc}$, thanks to Lemma 5.1.

Now, observe that the compatibility condition for the Poincaré system (5.10) is equivalent to

$$\begin{aligned} \partial _b \big (\omega (\partial _a)\cdot A\big ) - \partial _a \big (\omega (\partial _b) \cdot A \big ) = 0 \end{aligned}$$

for each index $1\le a,b \le n+k$. Invoking identity (5.17) once more, we can express

$$\begin{aligned} d(w\cdot A)(\partial _a, \partial _b) - \partial _b \big (\omega (\partial _a)\cdot A\big ) + \partial _a \big (\omega (\partial _b) \cdot A \big )=0. \end{aligned}$$

Therefore, it suffices to show that

5.19 $$\begin{aligned} d(w\cdot A)=0. \end{aligned}$$

Indeed, using the Pfaff system (5.9) solved above, we have

5.20 $$\begin{aligned} d(w\cdot A)= dw \cdot A - w \wedge dA = dw \cdot A - w \wedge (W\cdot A). \end{aligned}$$

However, the first structural equation (5.1) can be expressed as

$$\begin{aligned} dw^i = \sum _a \omega ^a \wedge \omega ^i_a = \sum _j \omega ^j \wedge \omega _j^i, \end{aligned}$$

since $w_\beta =0$ for any $n+1 \le \beta \le n+k$ by construction. Then

5.21 $$\begin{aligned} dw\cdot A = w\wedge (W\cdot A). \end{aligned}$$

Putting Eqs. (5.20)–(5.21) together, we deduce (5.19), which is equivalent to the compatibility condition for the Poincaré system (5.10). As a consequence, we find that $f\in W^{2,p}_\mathrm{loc}(V;\mathbb {R}^{n+k})$ solves Eq. (5.10).

**6.** With the solution to the Pfaff system (5.9) and the Poincaré system (5.10) associated with our isometric immersions problem, it remains to check that *f* is indeed the immersion for which we are seeking. To this end, we can define a new local moving frame $\{e_1, \ldots , e_{n+k}\}$ by

5.22 $$\begin{aligned} e_i := df(\partial _i), \qquad e_\alpha := \sum _b A^\alpha _b \partial _b. \end{aligned}$$

Then, following precisely the same arguments as those in pages 32–35 in [47], we can show that such a construction gives the correct normal bundle metric, normal affine connection, and second fundamental form.

Moreover, observe that $df= w\,\cdot A$ from Eq. (5.10), $A \in O(n+k)$ is a field of nonsingular matrices, and $\{\omega ^1, \ldots , \omega ^n\}$ are linearly independent, so that $df \ne 0$ in $W^{1,p}_\mathrm{loc}$. Since $p > n$, the Sobolev embedding yields that $df \ne 0$ almost everywhere, which verifies that *f* is indeed an immersion. The almost everywhere uniqueness of *f* follows from Lemma 5.1 and the $\mathbb {R}^{n+k}\rtimes O(n+k)$-symmetry of the Euclidean space.

**7.** It remains to globalize our arguments for simply connected manifolds. This follows from a standard argument in topology.

Given any two points $x \ne y \in M$, we connect them by a continuous curve (again since $g \in W^{1,p}_\mathrm{loc}\hookrightarrow C^0_\mathrm{loc}$ for $p>n$), denoted by $\gamma : [0,1]\mapsto M$ with $\gamma (0)=x$ and $\gamma (1)=y$. Let *f* be the $W^{2,p}_\mathrm{loc}$ isometric immersion in a neighborhood of *x*, whose existence is guaranteed by the preceding steps of the same proof. Cover curve $\gamma ([0,1])$ by finitely many charts $\{V_1,\ldots ,V_N\}$. By the uniqueness statements in Lemma 5.1, we can extend the isometric immersion *f* to $\bigcup _{i=1}^N V_i$, especially including a neighborhood of *y*.

Thus, it suffices to verify that the extension of *f* is independent of the choice of $\gamma$. Indeed, if $\eta :[0,1]\mapsto M$ is another continuous curve connecting *x* and *y*, by concatenating $\gamma$ with $\eta$, we can obtain a loop $L\subset M$. As *M* is simply connected, the restriction $f|_L$ is homotopic to a constant map so that $(f\circ \gamma )(1)=(f\circ \eta )(1)$. In this way, we have verified that *f* can be extended to a global isometric immersion of *M* into $\mathbb {R}^{n+k}$, provided that *M* is simply connected.

This completes the proof of Theorem 5.2.

As a corollary of Theorems 4.1 and 5.2, we can deduce the weak rigidity of isometric immersions.

#### Corollary 5.2

(Weak rigidity of isometric immersions) Let *M* be an *n*-dimensional simply connected Riemannian manifold with metric $g\in W^{1,p}_\mathrm{loc}$ for $p>n$. Suppose that $\{f^\epsilon \}$ is a family of isometric immersions of *M* into $\mathbb {R}^{n+k}$, uniformly bounded in $W^{2,p}_\mathrm{loc}(M; \mathbb {R}^{n+k})$, whose second fundamental forms and normal connections are $\{B^\epsilon \}$ and $\{\nabla ^{\perp ,\epsilon }\}$, respectively. Then, after passing to subsequences, $\{f^\epsilon \}$ converges to $\bar{f}$ weakly in $W_{\mathrm{loc}}^{2,p}$ which is still an isometric immersion $\bar{f}: (M,g) \hookrightarrow \mathbb {R}^{n+k}$. Moreover, the corresponding second fundamental form $\bar{B}$ is a limit point of $\{B^\epsilon \}$, and the corresponding normal connection $\overline{\nabla ^\perp }$ is a limit point of $\{\nabla ^{\perp ,\epsilon }\}$, both taken in the $L^p_\mathrm{loc}$ topology.

#### Proof

For a sequence $\{f^\epsilon \}$ of isometric immersions, uniformly bounded in $W^{2,p}_\mathrm{loc}(M; \mathbb {R}^{n+k})$, we can define the corresponding second fundamental forms and normal affine connections, denoted by $(B^\epsilon ,\nabla ^{\perp ,\epsilon })$.

By Theorem 5.2, $(B^\epsilon ,\nabla ^{\perp ,\epsilon })$ satisfy the GCR equations in the distributional sense and are uniformly bounded in the $L^p$ norm. Let $(\overline{B},\overline{\nabla ^\perp })$ be a weak limit of this family. Now, in view of Theorem 4.1 on the weak rigidity of the GCR equations, $(\overline{B},\overline{\nabla ^\perp })$ is still a solution to the GCR equations in the distributional sense. Thus, invoking Theorem 5.2 again, we can find a $W^{2,p}_\mathrm{loc}$ isometric immersion $\overline{f}$, for which $\overline{B}$ and $\overline{\nabla ^\perp }$ are its second fundamental form and normal connection, respectively. By the uniqueness of distributional limits, $\overline{f}$ must coincide with the weak limit of $\{f^\epsilon \}$.

Therefore, we have verified that the weak limit of a sequence of isometric immersions of manifold *M* is still an isometric immersion of *M*, with corresponding second fundamental form and normal connection. This completes the proof. $\square$

One natural question arises at this stage is about the criteria for the existence of global isometric immersions for a non-simply connected manifold *M* with $W^{1,p}_\mathrm{loc}$ metrics, especially for the case of the minimal target dimension (i.e., the Janet dimension). However, as far as we have known, it is still open, primarily owing to some topological obstructions to the GCR equations. Suppose that there is a loop $\gamma$ generating nontrivial homotopy on *M*, it is far from being clear if one can find a well-defined immersion along the entire loop. The problems on global isometric immersions/embeddings for general manifolds remain vastly open in the large. See Schoen–Yau [41] and Bryant–Griffith–Yang [4] for further discussions.

Therefore, the best we may say for non-simply-connected manifolds are the *local* versions of Theorem 5.2 and Corollaries 5.1 and 5.2.

## The Critical Case: $n=p=2$

Our main geometric result established in Sect. 5, i.e., Theorem 5.2, deals with the Riemannian manifolds with $W^{1,p}_\mathrm{loc}$ metrics for $p>n$. Even for the weak rigidity of the GCR equations, we still require $p>2$, as in Sect. 4. Therefore, $n=p=2$ becomes a critical case, from both the geometric perspectives and the PDE point of view. This is what we investigate in this section. We focus on a 2-dimensional manifold *M* with some Riemannian metric $g\in H^1_\mathrm{loc}$.

The difficulty lies in the insufficiency of regularity for certain Sobolev embeddings. Notice that, for $n=p=2$, the second fundamental forms and normal connections $(B^\epsilon ,\nabla ^{\perp ,\epsilon })$ are only uniformly bounded in $L^2_\mathrm{loc}$, so the quadratic nonlinearities in the GCR equations are at most bounded in $L^1_\mathrm{loc}$. However, $L^1_\mathrm{loc}$ cannot be embedded into $H^{-1}_\mathrm{loc}$. This can be seen via the dual spaces, since $H^1(\mathbb {R}^2)$ is only continuously, but not compactly, embedded into $BMO (\mathbb {R}^2)$, which is the space of functions of bounded mean oscillations; moreover, $BMO (\mathbb {R}^2)$ is strictly contained in $L^\infty (\mathbb {R}^2)$. A standard example for $f \in H^{1}(\mathbb {R}^2) \setminus L^\infty (\mathbb {R}^2)$ is $f(x)=\log \log (|x|^{-1})\chi _{B_1(0)}$.

However, if the codimension of the immersion is 1, i.e., the surface *M* is immersed into $\mathbb {R}^3$, we can still obtain the weak rigidity. For this purpose, we need the corresponding critical case of Theorem 3.3, in which $\{d\omega ^\epsilon \}$ and $\{\delta \tau ^\epsilon \}$ are contained in compact subsets of $W^{-1,1}_\mathrm{loc}$ spaces. This has been treated in Conti–Dolzmann–Müller in [12].

We now state and prove a slight variant of the critical case result in [12], formulated for global differential forms on the Riemannian manifolds. This can be achieved by combining the global arguments on the manifolds developed above with the arguments in [12]; thus, its proof will be only sketched briefly.

### Theorem 6.1

Let (*M*, *g*) be an *n*-dimensional manifold. Let $\{\omega ^\epsilon \}, \{\tau ^\epsilon \}\subset L^2_\mathrm{loc}(M;\bigwedge ^q T^*M)$ be two families of differential *q*-forms, for $0\le q\le n$. Suppose that

(i) $\omega ^\epsilon \rightharpoonup \overline{\omega }$ weakly in $L^2$, and $\tau ^\epsilon \rightharpoonup \overline{\tau }$ weakly in $L^2$;
(ii) There are compact subsets of the corresponding Sobolev spaces, $K_d$ and $K_\delta$, such that
  $$\begin{aligned} {\left\{ \begin{array}{ll} \{d\omega ^\epsilon \}\subset K_d \Subset W^{-1,1}_\mathrm{loc}(M;\bigwedge ^{q+1}T^*M),\\ \{\delta \tau ^\epsilon \}\subset K_\delta \Subset W^{-1,1}_\mathrm{loc}(M;\bigwedge ^{q-1}T^*M)\mathrm{;} \end{array}\right. } \end{aligned}$$
(iii) $\{\langle \omega ^\epsilon ,\tau ^\epsilon \rangle \}$ is equi-integrable.

Then $\langle \omega ^\epsilon , \tau ^\epsilon \rangle$ converges to $\langle \overline{\omega }, \overline{\tau }\rangle$ in the sense of distributions:

$$\begin{aligned} \int _M \langle \omega ^\epsilon , \tau ^\epsilon \rangle \psi \, \mathrm{d}V_g \longrightarrow \int _M \langle \overline{\omega }, \overline{\tau }\rangle \psi \, \mathrm{d}V_g \qquad \hbox {for any }\psi \in C^\infty _{c}(M). \end{aligned}$$

### Proof

First of all, let us make two reductions. First, as in the proof for Theorem 3.3, it suffices to prove for compact orientable manifold *M*; Second, without loss of generality, we may take $\bar{\omega }=\bar{\tau }=0$.

Next we perform a truncation to $\{\omega ^\epsilon \}$ and $\{\tau ^\epsilon \}$. By assumption (i), sequence $\{|\omega ^\epsilon |^2\}$ is weakly convergent in $L^1$. Hence, applying Chacon’s biting lemma (*cf.* [2]), one can find subsets $K_\epsilon \subset M$ such that $|K_\epsilon |_{g}:=\int _M \chi _{K_{\epsilon }}dV_g\rightarrow 0$ and $\{|\omega ^\epsilon |^2 \chi _{M\setminus K_\epsilon }\}$ is equi-integrable. We define the truncated differential forms $\tilde{\omega }^\epsilon :=\omega ^\epsilon \chi _{M\setminus K_\epsilon }$. Similarly, we take $\tilde{K}_\epsilon \subset M$ such that $|\tilde{K}_\epsilon |_g \rightarrow 0$ and $\{|\tau ^\epsilon |^2 \chi _{M\setminus \tilde{K}_\epsilon }\}$ is equi-integrable to obtain the truncated forms $\tilde{\tau }^\epsilon :=\tau ^\epsilon \chi _{M\setminus \tilde{K}_\epsilon }$.

Now, let us measure the $L^1$ difference of $\omega ^\epsilon$ and $\tilde{\omega }^\epsilon$. By the Cauchy–Schwarz inequality,

$$\begin{aligned} \Vert \tilde{\omega }^\epsilon -\omega ^\epsilon \Vert _{L^1(M;\bigwedge ^q T^*M)} = \Vert \omega ^\epsilon \Vert _{L^1(K_\epsilon )} \le \sqrt{|R_\epsilon |_g} \Vert \omega ^\epsilon \Vert _{L^2(M;\bigwedge ^q T^*M)} \rightarrow 0, \end{aligned}$$

since $\{\omega ^\epsilon \}$ is uniformly bounded in $L^2$ owing to assumption (i). Moreover, by assumption (ii), $\Vert d\tilde{\omega }^\epsilon \Vert _{W^{-1,1}(M;\bigwedge ^{q+1}T^*M)} \rightarrow 0$. Analogously, we find that $\Vert \tilde{\tau }^\epsilon -\tau ^\epsilon \Vert _{L^1(M;\bigwedge ^q T^*M)}\rightarrow 0$ and $\Vert \delta \tilde{\tau }^\epsilon \Vert _{W^{-1,1}(M;\bigwedge ^{q-1}T^*M)}\rightarrow 0$. Moreover, we note that

6.1 $$\begin{aligned} \langle \omega ^\epsilon , \tau ^\epsilon \rangle - \langle \tilde{\omega }^\epsilon , \tilde{\tau }^\epsilon \rangle \rightarrow 0, \end{aligned}$$

in view of the equi-integrability assumption (iii) and $|K_\epsilon \cup \tilde{K}_\epsilon |_g \rightarrow 0$. By the Dunford–Pettis theorem and assumption (iii), we know that $\{\langle \omega ^\epsilon ,\tau ^\epsilon \rangle \}$ is weakly pre-compact in $L^1$. Hence, Eq. (6.1) implies that $\{\langle \omega ^\epsilon ,\tau ^\epsilon \rangle \}$ and $\{\langle \tilde{\omega }^\epsilon , \tilde{\tau }^\epsilon \rangle \}$ have the same distributional limits.

Thus, it remains to show that $\langle \tilde{\omega }^\epsilon , \tilde{\tau }^\epsilon \rangle \rightarrow 0$ in the distributional sense. In fact, by the Lipschitz truncation argument in [12], $d \tilde{\omega }^\epsilon$ and $\delta \tilde{\tau }^\epsilon$ converge to 0 in $H^{-1}_\mathrm{loc}$, after passing to subsequences. Therefore, we can conclude the proof from the intrinsic div-curl lemma, i.e., Theorem 3.3. $\square$

### Remark 6.1

In the proof of Theorem 6.1, the technique of Lipschitz truncation has played an important role, which reduces the div-curl lemma from the critical case to the usual case, where the target spaces are reflexive (*cf.* Theorem 3.3). Such a technique depends explicitly on the geometry of the underlying manifolds.

We remark that the endpoint case of the intrinsic div-curl lemma, i.e., Theorem 6.1, can also be generalized in a similar manner as for Theorem 3.4.

### Theorem 6.2

Let (*M*, *g*) be an *n*-dimensional manifold. Let $\{\omega ^\epsilon \}\subset L^r_\mathrm{loc}(M;\bigwedge ^q T^*M)$ and $\{\tau ^\epsilon \}\subset L^s_\mathrm{loc}(M;\bigwedge ^q T^*M)$ be two families of differential *q*-forms, for $0\le q\le n$, $1<r,s<\infty$, and $\frac{1}{r}+\frac{1}{s}=1$. Suppose that

(i) $\omega ^\epsilon \rightharpoonup \overline{\omega }$ weakly in $L^r$, and $\tau ^\epsilon \rightharpoonup \overline{\tau }$ weakly in $L^s$ as $\epsilon \rightarrow 0$;
(ii) There are compact subsets of the corresponding Sobolev spaces, $K_d$ and $K_\delta$, such that
  $$\begin{aligned} {\left\{ \begin{array}{ll} \{d\omega ^\epsilon \}\subset K_d \Subset W^{-1,1}_\mathrm{loc}(M;\mathop {\bigwedge }\nolimits ^{q+1}T^*M),\\ \{\delta \tau ^\epsilon \}\subset K_\delta \Subset W^{-1,1}_\mathrm{loc}(M;\mathop {\bigwedge }\nolimits ^{q-1}T^*M); \end{array}\right. } \end{aligned}$$
(iii) $\{\langle \omega ^\epsilon ,\tau ^\epsilon \rangle \}$ is equi-integrable.

Then $\langle \omega ^\epsilon , \tau ^\epsilon \rangle$ converges to $\langle \overline{\omega }, \overline{\tau }\rangle$ in the sense of distributions:

$$\begin{aligned} \int _M \langle \omega ^\epsilon , \tau ^\epsilon \rangle \psi \, \mathrm{d}V_g \longrightarrow \int _M \langle \overline{\omega }, \overline{\tau }\rangle \psi \, \mathrm{d}V_g \qquad \hbox {for any }\psi \in C^\infty _{c}(M). \end{aligned}$$

The proof of Theorem 6.2 follows directly by combining the argument for Theorem 3.4 in the appendix with the proof for Theorem 6.1.

After introducing the intrinsic div-curl lemmas in the critical case, we can now establish the global weak rigidity of isometric immersions for a surface into $\mathbb {R}^3$:

### Theorem 6.3

Let *M* be a 2-dimensional, simply connected surface, and let *g* be a metric in $H^{1}_\mathrm{loc}$. If $\{f^\epsilon \}$ is a family of $H^{2}_\mathrm{loc}$ isometric immersions of *M* into $\mathbb {R}^3$ such that the corresponding second fundamental forms $\{B^\epsilon \}$ are uniformly bounded in $L^2$. Then, after passing to subsequences, $\{f^\epsilon \}$ converges to $\bar{f}$ weakly in $H^2_\mathrm{loc}$ which is still an isometric immersion $\bar{f}:(M,g) \hookrightarrow \mathbb {R}^3$. Moreover, the corresponding second fundamental form $\bar{B}$ is a weak limit of $\{B^\epsilon \}$ in $L^2_\mathrm{loc}$.

### Proof

We divide the proof into three steps.

**1.** Since the codimension of the immersions $\{f^\epsilon \}$ is 1, the normal affine connections are trivial. This is because, in the Ricci equation:

$$\begin{aligned} \langle [S_\eta , S_\xi ]X,Y\rangle = R^\perp (X,Y,\eta ,\xi ), \end{aligned}$$

we can only take the normal vector fields $\eta$ and $\xi$ to be linearly dependent, as the fiber for the normal bundle is simply $\mathbb {R}$. Then both sides are equal to zero, by the definition of $R^\perp$ and the Lie bracket. Now we look at the Codazzi equation. In the second formulation for Theorem 4.1, we have obtained Eq. (4.20), which is

$$\begin{aligned} 0=d\Big (\Omega ^{(B)}_{Z,\eta }\Big )(X,Y) + \sum _\beta \Big \langle V^{(\nabla ^\perp )}_{\eta ,\beta }(X,Y),\Omega ^{(B)}_{Z,\beta }\Big \rangle + E(B). \end{aligned}$$

However, since $\nabla ^\perp$ is trivial, the equation is reduced to

6.2 $$\begin{aligned} d(\Omega ^{(B)}_{Z,\eta })(X,Y) = -E(B), \end{aligned}$$

where *E*(*B*) is linear in *B*. We notice that the Codazzi equation in the critical case becomes linear, as the quadratic nonlinearities are absent, so that we can pass to the weak limits.

**2.** It remains to prove the weak rigidity of the Gauss equation:

$$\begin{aligned} \langle B^\epsilon (Y,W), B^\epsilon (X,Z) \rangle -\langle B^\epsilon (X,W), B^\epsilon (Y,Z)\rangle = R(X,Y,Z,W). \end{aligned}$$

By assumption, $\{B^\epsilon \}$ is uniformly bounded in $L^2_\mathrm{loc}$, so that $R \in L^1_\mathrm{loc}(M; \bigotimes ^4 T^*M)$ is a fixed function, by the Cauchy–Schwarz inequality. It follows that

$$\begin{aligned}&\int _M \Big |\langle B^\epsilon (Y,W), B^\epsilon (X,Z) \rangle - \langle B^\epsilon (X,W), B^\epsilon (Y,Z)\rangle \Big |\chi _A\, \mathrm{d}V_g \\&\quad \le \int _M \chi _A|R(X,Y,Z,W)|\, \mathrm{d}V_g \rightarrow 0 \qquad \text { as } |A|_g \rightarrow 0, \end{aligned}$$

i.e., the set of quadratic nonlinearities $\{\langle B^\epsilon (Y,W), B^\epsilon (X,Z) \rangle - \langle B^\epsilon (X,W), B^\epsilon (Y,Z)\rangle \}$ is equi-integrable. Here it is crucial that *R* is a locally integrable function, not just a Radon measure.

**3.** Now, we invoke again the arguments in Sect. 4 to analyze the div-curl structure of the Gauss equation. First, recall our definition for the tensor field $V^{(B)}_{Z,\eta }: \Gamma (TM)\times \Gamma (TM)\mapsto \Gamma (TM)$ and the 1-form $\Omega ^{(B)}_{Z,\eta } \in \Omega ^1(M)=\Gamma (T^*M)$. We set

$$\begin{aligned} {\left\{ \begin{array}{ll} V^{(B)}_{Z,\eta }(X,Y):= B(X,Z,\eta )Y-B(Y,Z,\eta )X, \\ \Omega ^{(B)}_{Z,\eta }:= -B(\bullet , Z, \eta ). \end{array}\right. } \end{aligned}$$

Then the first formulation in Sect. 4 leads to

6.3 $$\begin{aligned} {\left\{ \begin{array}{ll} \mathrm{div}\big (V^{(B)}_{Z,\eta }(X,Y)\big )= YB(X,Z,\eta ) - X B(Y,Z,\eta ),\\ d \big (\Omega ^{(B)}_{Z,\eta }\big )(X,Y) = YB(X,Z,\eta ) - XB(Y,Z,\eta ) + B([X,Y],Z,\eta ), \end{array}\right. } \end{aligned}$$

and the second formulation in Sect. 4 enables us to recast the Gauss equation into the following form:

$$\begin{aligned} \sum _\eta \langle V^{(B)}_{Z,\eta }(X,Y), \Omega ^{(B)}_{W,\eta } \rangle =R(X,Y,Z,W). \end{aligned}$$

Since $\{B^\epsilon \}$ is uniformly bounded in $L^2_\mathrm{loc}$, we can deduce from the equations in (6.3) that $\{\delta V^{(B^\epsilon )}\}$ and $\{d\Omega ^{(B^\epsilon )}\}$ are relatively compact in $W^{-1,1}_\mathrm{loc}$. Now we can employ the critical case of our intrinsic div-curl lemma, Theorem 6.2, to conclude that

6.4 $$\begin{aligned}&\langle B^\epsilon (X,W), B^\epsilon (Y,Z)- \langle B^\epsilon (Y,W), B^\epsilon (X,Z) \rangle \nonumber \\&\quad \longrightarrow \langle \overline{B}(X,W), \overline{B}(Y,Z) - \langle \overline{B}(Y,W), \overline{B}(X,Z) \rangle \end{aligned}$$

in the distributional sense, where $\overline{B}$ is the $L^2_\mathrm{loc}$ weak limit of $\{B^\epsilon \}$ up to subsequences. From here, we conclude the weak rigidity of the Gauss equation. Therefore, the proof is complete. $\square$

## Further Results and Remarks

In this section, we first provide a proof of the weak rigidity of the Cartan formalism. Then we extend the weak rigidity theory to allow manifolds $(M, g^\epsilon )$ with metrics $\{g^\epsilon \}$ converging to *g* in $W^{1,p}_\mathrm{loc}, p>n$, instead of the fixed manifold (*M*, *g*) with fixed metric *g*.

### Weak Rigidity of the GCR Equations and Isometric Immersions Revisited

We now provide a proof of the weak rigidity of the Cartan formalism, which leads to the weak rigidity of the GCR equations and isometric immersions, in view of the equivalence between the Cartan formalism, the GCR equations, and isometric immersions established in Sect. 5. Therefore, we also provide an alternative proof of Theorem 4.1 and Corollary 5.2. The arguments are summarized as follows:

#### Alternative Proof of Theorem 4.1 and Corollary 5.2

Observe that all the nonlinear terms in the Cartan formalism are contained in the second structural equation, i.e.,

7.1 $$\begin{aligned} dW=-W\wedge W. \end{aligned}$$

Thus it suffices to establish the weak rigidity of (7.1).

Indeed, consider the family of connection forms $\{W^\epsilon \}$ associated with $\{(B^\epsilon ,\nabla ^{\perp ,\epsilon })\}$ in the Cartan formalism for isometric immersions; see Sect. 5. As $\{(B^\epsilon ,\nabla ^{\perp ,\epsilon })\}$ is uniformly bounded in $L^p_{\text {loc}}$, the same holds for $\{W^\epsilon \}$. Also, recall that $W^\epsilon$ is a Lie algebra-valued 1-form, i.e., it is an element of $\Omega ^1(\mathfrak {so}(n+k)) \cong \mathfrak {so}(n+k) \bigotimes \Omega ^1(M)$. Throughout this proof, the Hodge star $*$ is always understood as taken with respect to the $\Omega ^q(M)$ factor of $\mathfrak {so}(n+k) \bigotimes \Omega ^q(M)$. We see from the definition of Sobolev spaces of tensor fields in Sect. 2 that $*$ is an isometry between $W^{k,p}(M; \bigwedge ^q T^*M)$ and $W^{k,p}(M; \bigwedge ^{n-q} T^*M)$ for any $k, p, \hbox { and } q$.

Next, we take an arbitrary test differential form $\eta \in C^\infty _c(M; \bigwedge ^{n-2} T^*M)$, which is independent of $\epsilon$ and has a trivial $\mathfrak {so}(n+k)$-component. Eq. (7.1) can then be recast as

7.2 $$\begin{aligned} dW^\epsilon \wedge \eta = - W^\epsilon \wedge W^\epsilon \wedge \eta . \end{aligned}$$

Define $$V^\epsilon := *(W^\epsilon \wedge \eta ) \in \Omega ^{1}(\mathfrak {so}(n+k))$$. Using $$\delta = \pm *d *$$, $$**= \pm \mathrm{id}$$, and $$\langle \alpha ,\beta \rangle = \alpha \wedge *\beta $$ for differential forms $$\alpha $$ and $$\beta $$ of the same order (*cf.* Sect. 2), we obtain the next two equalities:

7.3 $$\begin{aligned}&\langle dW^\epsilon , *\eta \rangle = \pm \langle W^\epsilon , V^\epsilon \rangle , \end{aligned}$$

7.4 $$\begin{aligned}&*\delta V^\epsilon = \pm (*W^\epsilon ) \wedge V^\epsilon \pm W^\epsilon \wedge d\eta . \end{aligned}$$

For our purpose, we bound only for relevant geometric quantities in some $W^{k,p}_\mathrm{loc}$ so that there is no need to keep track of the specific signs: Instead, we just write ± in suitable places.

With these, we now analyze the weak rigidity of Eq. (7.1). As $\eta \in C^\infty _c$, and the Hodge star $*$ is isometric, the Cauchy–Schwarz inequality gives that $\{\langle W^\epsilon , V^\epsilon \rangle \}$ and $\{(*W^\epsilon ) \wedge V^\epsilon \}$ are uniformly bounded in $L^{p/2}_{\mathrm{loc}}$ for $p>2$. Also, $\{W^\epsilon \wedge d\eta \}$ is uniformly bounded in $L^p_\mathrm{loc}$ for $p>2$. Thus, in view of the Sobolev embeddings and Eqs. (7.3)–(7.4), we find that $\{dW^\epsilon \}$ and $\{\delta V^\epsilon \}$ are pre-compact in $W^{-1, r}_{\mathrm{loc}}$, with $1<r<2$. On the other hand, $\{dW^\epsilon \}$ and $\{\delta V^\epsilon \}$ are uniformly bounded in $W^{-1, p}_\mathrm{loc}$ for $p>2$. Thus, by interpolation, the curl (i.e., *d*) of $\{W^\epsilon \}$ and the divergence (i.e., $\delta$ modulo the sign) of $\{V^\epsilon \}$ are pre-compact in $H^{-1}_\mathrm{loc}$.

Then, employing the geometric div-curl lemma (i.e., Theorem 3.3), $\langle W^\epsilon , V^\epsilon \rangle = *(W^\epsilon \wedge *V^\epsilon )$ converges to $\langle W, V\rangle$ in the distributional sense, where *W* and *V* are the weak $L^p_\mathrm{loc}$ limits of $\{W^\epsilon \}$ and $\{V^\epsilon \}$, respectively. Moreover, $V = *(W \wedge \eta )$. Substituting them into Eq. (7.2), we have

7.5 $$\begin{aligned} dW \wedge \eta = - W\wedge W \wedge \eta . \end{aligned}$$

Then, by the arbitrariness of $\eta \in C^\infty _c(M; \bigwedge ^{n-2} T^*M)$, we conclude the weak rigidity of the second structural equation (7.1).

Therefore, in view of the equivalence of the Cartan formalism, the GCR equations, and the isometric immersions of Riemannian manifolds (*cf.* Theorem 5.2, Theorem 4.1, and Corollary 5.2), the proof is now complete. $\square$

We remark that the above proof lies in the same spirit as in Chen–Slemrod–Wang [8] for the GCR equations. Related arguments are also present in [1, 11, 18, 19, 33–35, 45, 46] and the references cited therein.

### Weak Rigidity of Isometric Immersions with Different Metrics

We now develop an extension of the weak rigidity theory of the GCR equations and isometric immersions established above. In the earlier sections, we have analyze the isometric immersions of a *fixed* Riemannian manifold. In particular, despite the change of second fundamental forms and normal connections as we shift between distinctive immersions, metric $g\in W^{1,p}_{\text {loc}}$ is always fixed. In what follows, we generalize the weak rigidity results (*cf.* Theorem 4.1 and Corollary 5.2) to the Riemannian manifolds with unfixed metrics $\{g^\epsilon \}$.

More precisely, we establish the following theorem.

#### Theorem 7.1

Let $(M, g^\epsilon )$ be a sequence of *n*-dimensional simply connected Riemannian manifolds with metrics $\{g^\epsilon \}\subset W^{1,p}_{\text {loc}}(M;\text {Sym}^2T^*M)$ converging strongly in $W^{1,p}_{\text {loc}}$ for $p>n$. Suppose that there exists a family of corresponding isometric immersions of $(M,g^\epsilon )$ converging weakly in $W^{2,p}_{\text {loc}}(M;\mathbb {R}^{n+k})$, denoted by $\{f^\epsilon \}$, with second fundamental forms $\{B^\epsilon \}$ and normal connections $\{\nabla ^{\perp ,\epsilon }\}$. Then, after passing to subsequences, $\{f^\epsilon \}$ converges weakly to an isometric immersion $\bar{f}$ of (*M*, *g*) in $W^{2,p}_\mathrm{loc}$, where *g* is the $W^{1,p}_{\text {loc}}$ limit of $\{g^\epsilon \}$. Moreover, the second fundamental form $\overline{B}$ and normal connection $\overline{\nabla ^\perp }$ of immersion $\bar{f}$ coincide with the corresponding subsequential weak $L^p_{\text {loc}}$-limits of $\{B^\epsilon \}$ and $\{\nabla ^{\perp ,\epsilon }\}$.

#### Proof

In this proof, we write the inner product of *X* and *Y* induced by $g^\epsilon$ by $g^\epsilon (X,Y)$, and the previous notation $\langle \cdot ,\cdot \rangle$ is reserved for the paring of $(TM, T^*M)$. Moreover, $g_0$ always denotes the Euclidean inner product in $\mathbb {R}^{n+k}$. We also write $\nabla ^\epsilon$ and $R^\epsilon$ for the Levi–Civita connection and Riemann curvature tensor, respectively, associated with $g^\epsilon$. We divide the proof into four steps.

**1.** First of all, we again rewrite the GCR equations as in the first and second formulation in Sect. 4 (*cf.* Lemmas 4.1–4.2). Indeed, defining the vector fields *V* and 1-forms $\Omega$ as follows:

$$\begin{aligned} \begin{array}{lll} &{}V^{(B^\epsilon )}_{Z,\eta }(X,Y)= g_0(B^\epsilon (X,Z),\eta ) Y - g_0(B^\epsilon (Y,Z),\eta )X, &{}\qquad \Omega ^{(B^\epsilon )}_{Z,\eta } = -g_0(B^\epsilon (\bullet ,Z),\eta ), \\ &{}V^{(\nabla ^{\perp , \epsilon })}_{\xi ,\eta } (X,Y) = g_0(\nabla ^{\perp , \epsilon }_Y\xi ,\eta )X-g_0(\nabla ^{\perp , \epsilon }_X\xi , \eta )Y, &{}\qquad \Omega ^{(\nabla ^{\perp ,\epsilon })}_{\xi ,\eta } = g_0 (\nabla ^{\perp , \epsilon }_\bullet \xi ,\eta ), \end{array} \end{aligned}$$

we can establish the equivalence of the GCR equations with the following three expressions:

7.6 $$\begin{aligned}&\sum _\eta \Big \langle V^{(B^\epsilon )}_{W,\eta }(X,Y),\Omega ^{(B^\epsilon )}_{Z,\eta } \Big \rangle = R^\epsilon (X,Y,W,Z), \end{aligned}$$

7.7 $$\begin{aligned}&\quad d\Omega ^{(B^\epsilon )}_{Z,\eta }(X,Y) + \sum _\beta \Big \langle V^{(\nabla ^{\perp ,\epsilon })}_{\eta ,\beta }(X,Y),\Omega ^{(B^\epsilon )}_{Z,\beta }\Big \rangle + g_0\big (B^\epsilon (Y,\nabla ^\epsilon _X Z),\eta \big )\nonumber \\&\qquad - g_0\big (B^\epsilon (X,\nabla ^\epsilon _YZ),\eta \big ) =0, \end{aligned}$$

7.8 $$\begin{aligned}&d\Omega ^{(\nabla ^{\perp ,\epsilon })}_{\xi ,\eta }(X,Y) + \sum _\beta \Big \langle V^{(\nabla ^{\perp ,\epsilon })}_{\eta ,\beta }(X,Y), \Omega ^{(\nabla ^{\perp ,\epsilon })}_{\xi ,\beta } \Big \rangle = \sum _Z\Big \langle V^{(B^\epsilon )}_{Z,\xi }(X,Y), \Omega ^{(B^\epsilon )}_{Z,\eta } \Big \rangle .\nonumber \\ \end{aligned}$$

The derivations of these identities are the same as in Lemma 4.2.

**2.** We now analyze the mode of convergence for each term. Since $\{f^\epsilon \}$ is weakly convergent in the $W^{2,p}_{\text {loc}}$ norm (hence $\{B^\epsilon \}$ and $\{\nabla ^{\perp ,\epsilon }\}$ are uniformly bounded in $L^p_{\text {loc}}$), we know that $\big \{V^{(B^\epsilon )}_{Z,\eta }(X,Y), V^{(\nabla ^{\perp ,\epsilon })}_{\xi ,\eta }(X,Y)\big \} \subset L^p_{\text {loc}}(TM)$ and $\big \{\Omega ^{(B^\epsilon )}_{Z,\eta }, \Omega ^{(\nabla ^{\perp ,\epsilon })}_{\xi ,\eta }\big \} \subset L^p_{\text {loc}}(T^*M)$ are also uniformly bounded. Moreover, from the assumption that $\{g^\epsilon \}$ is strongly convergent in $W^{1,p}_{\text {loc}}$, the Levi–Civita connections $\nabla ^\epsilon : \Gamma (TM) \times \Gamma (TM) \rightarrow \Gamma (TM)$ are strongly convergent in $L^p_{\text {loc}}$. This can be seen either by the local expression

$$\begin{aligned} (\Gamma ^\epsilon )^i_{jk}:={g^\epsilon }(\nabla _{\partial _i}\partial _j,\partial _k) = \frac{1}{2}(g^\epsilon )^{il}\big (\partial _j g^\epsilon _{kl} +\partial _k g^\epsilon _{jl}-\partial _l g^\epsilon _{jk} \big ), \end{aligned}$$

or from the *Koszul formula*:

7.9 $$\begin{aligned} 2g^\epsilon (\nabla ^\epsilon _XY,Z)&= Xg^\epsilon (Y,Z) + Yg^\epsilon (X,Z) - Zg^\epsilon (X,Y)\nonumber \\&\ \quad + g^\epsilon ([X,Y],Z) - g^\epsilon ([X,Z],Y) - g^\epsilon ([Y,Z],X), \end{aligned}$$

together with the compact embedding $W^{1,p}_{\text {loc}}(\mathbb {R}^n) \hookrightarrow L^\infty _{\text {loc}}(\mathbb {R}^n)$ for $p>n$. As a consequence, since $g_0\big (B^\epsilon (Y,\nabla ^\epsilon _X Z),\eta \big ) - g_0\big (B^\epsilon (X,\nabla ^\epsilon _YZ),\eta \big )$ is a product of strongly $L^p_{\text {loc}}$ convergent and weakly $L^p_{\text {loc}}$ convergent (equivalently, $L^p_{\text {loc}}$ bounded) tensors for $p>n$, we can pass to the limit, i.e., it converges to $g_0\big (\overline{B}(Y,\nabla _XZ),\eta \big )-g_0\big (\overline{B}(X,\nabla _YZ),\eta \big )$ modulo subsequences.

**3.** Now let us invoke the analogous equations to the first formulation (Lemma 4.1):

$$\begin{aligned}&\mathrm{div}\big (V^{(B^\epsilon )}_{Z,\eta }(X,Y)\big ) = Yg_0(B^\epsilon (X,Z),\eta ) - Xg_0(B^\epsilon (Y,Z),\eta ), \\&\mathrm{div}\big (V^{(\nabla ^{\perp ,\epsilon })}_{\xi ,\eta }(X,Y)\big ) =-Yg_0 (\nabla ^{\perp ,\epsilon }_X\xi ,\eta ) +Xg_0 (\nabla ^{\perp ,\epsilon }_Y\xi ,\eta ). \end{aligned}$$

Again, by the uniform bounds for $\{B^\epsilon \}$ and $\{\nabla ^{\perp ,\epsilon }\}$ in $L^p_{\text {loc}}$, we know that $\big \{\mathrm{div}\big (V^{(B^\epsilon )}_{Z,\eta }(X,Y)\big )\big \}$ and $\big \{\mathrm{div}\big (V^{(\nabla ^{\perp ,\epsilon })}_{\xi ,\eta }(X,Y)\big )\big \}$ are uniformly bounded in $W^{-1,p}_{\text {loc}}$, where $p > n \ge 2$. On the other hand, since

$$\begin{aligned}&d\Omega ^{(B^\epsilon )}_{Z,\eta }(X,Y) = Y g_0(B^\epsilon (X,Z),\eta )- Xg_0(B^\epsilon (Y,Z),\eta ) + g_0( B^\epsilon ([X,Y],Z),\eta ),\\&d\Omega ^{(\nabla ^{\perp ,\epsilon })}_{\xi ,\eta }(X,Y) = -Yg_0 (\nabla ^{\perp ,\epsilon }_X\xi ,\eta ) +Xg_0 (\nabla ^{\perp ,\epsilon }_Y\xi ,\eta ) - g_0(\nabla ^{\perp ,\epsilon }_{[X,Y]}\xi ,\eta ), \end{aligned}$$

by substituting the left-hand sides into (7.6)–(7.8) and applying the Hölder inequality to the suitable terms, we find that $\big \{\mathrm{div}\big (V^{(B^\epsilon )}_{Z,\eta }(X,Y)\big )\big \}$, $\big \{\mathrm{div}\big (V^{(\nabla ^{\perp ,\epsilon })}_{\xi ,\eta }(X,Y)\big )\big \}$, $\big \{d\Omega ^{(B^\epsilon )}_{Z,\eta }\big \}$, and $\big \{d\Omega ^{(\nabla ^{\perp ,\epsilon })}_{\xi ,\eta }\big \}$ are uniformly bounded in $L^{p/2}_{\text {loc}}$ so that, by interpolation, they are pre-compact in $H^{-1}_{\text {loc}}$.

**4.** The arguments in Step 3 enable us to apply the geometric div-curl lemma (as in Theorem 3.3), which leads to the following convergence in the distributional sense:

$$\begin{aligned}&\langle V^{(\nabla ^{\perp ,\epsilon })}_{\eta ,\beta }(X,Y),\Omega ^{(B^\epsilon )}_{Z,\beta }\rangle \longrightarrow \langle V^{(\overline{\nabla ^\perp })}_{\eta ,\beta }(X,Y), \Omega ^{(\overline{B})}_{Z,\beta }\rangle ,\\&\langle V^{(B^\epsilon )}_{W,\eta }(X,Y),\Omega ^{(B^\epsilon )}_{Z,\eta } \rangle \longrightarrow \langle V^{(\overline{B})}_{W,\eta }(X,Y), \Omega ^{(\overline{B})}_{Z,\eta } \rangle ,\\&\langle V^{(\nabla ^{\perp ,\epsilon })}_{\eta ,\beta }(X,Y), \Omega ^{(\nabla ^{\perp ,\epsilon })}_{\xi ,\beta } \rangle \longrightarrow \langle V^{(\overline{\nabla ^\perp })}_{\eta ,\beta }(X,Y), \Omega ^{(\overline{\nabla ^\perp })}_{\xi ,\beta }\rangle ,\\&\langle V^{(B^\epsilon )}_{Z,\xi }(X,Y), \Omega ^{(B^\epsilon )}_{Z,\eta } \rangle \longrightarrow \langle V^{(\overline{B})}_{Z,\xi }(X,Y), \Omega ^{(\overline{B})}_{Z,\eta }\rangle , \end{aligned}$$

where $(\overline{B}, \overline{\nabla ^\perp })$ are the subsequential weak limits of $\{(B^\epsilon , \nabla ^{\perp ,\epsilon })\}$. Therefore, we conclude that $(\overline{B}, \overline{\nabla ^\perp })$ satisfy the GCR equations in the distributional sense with respect to metric *g*, i.e., the subsequential strong $W^{1,p}_{\text {loc}}$ limit of $\{g^\epsilon \}$. Furthermore, as $f^\epsilon$ converges to $\bar{f}$ weakly in $W^{2,p}_{\text {loc}}(M, \mathbb {R}^{n+k})$, Corollary 5.2 implies that $\bar{f}$ is an isometric immersion with respect to the limiting metric *g*, and its second fundamental form and normal connection coincide with $\overline{B}$ and $\overline{\nabla ^\perp }$, respectively. Therefore, the proof is complete. $\square$

We also refer the reader to some recent results on the compactness of $W^{2,p}$ immersions of *n*-dimensional manifolds for $p>n$ with appropriate *gauges* for the case $n=2$ in Langer [29] and the higher dimensional case in Breuning [3].

## Appendix: Proof of Theorem 3.4

In this appendix, we give a proof for the intrinsic div-curl lemma, i.e., Theorem 3.4, on Riemannian manifolds. Some arguments for the proof are motivated from the work by Robbin–Rogers–Temple [40], in which a similar result has been established for the *local differential forms on the flat Euclidean spaces* $\mathbb {R}^n$; also see Sect. 5 in Kozono–Yanagisawa [28].

### Proof of Theorem 3.4

We divide the proof into five steps.

**1.** It suffices to prove Theorem 3.4 for closed, orientable manifolds. Indeed, by a partition of unity argument and assigning an orientation to each chart, it suffices to prove only for orientable manifolds. Now, consider an orientable manifold *M* which is not necessarily compact. Fix any test function $\psi \in C^\infty _c(M)$, and then choose $\phi \in C^\infty _c(M)$ such that $\phi |_{\mathrm{supp}(\psi )}\equiv 1$. For any differential form $\alpha$ on a compact subset of *M*, we can choose a compact oriented submanifold $\widetilde{M}\subset M$ with $supp(\phi )$ as its interior, and denote by $\widetilde{\alpha }=\alpha \phi$ as the extension-by-zero of $\alpha$ outside $\widetilde{M}$. Then it suffices to prove Theorem 3.4 for any compact orientable manifold $\widetilde{M}$, since, if the assertion holds for $\langle {\omega }^\epsilon , {\tau }^\epsilon \rangle$ on $\widetilde{M}$, then the theorem also holds for $\langle \omega ^\epsilon , \tau ^\epsilon \rangle$ on *M* in the distributional sense for any test function $\psi$ with $supp(\psi )\subset \widetilde{M}$.

**2.** We can now assume that *M* is a closed orientable manifold. Thus, Theorem 3.2 holds for the Laplace–Beltrami operator $\Delta =d\circ \delta + \delta \circ d$ on *M*. Then

A.1 $$\begin{aligned} \omega ^\epsilon= & {} \pi _H \omega ^\epsilon + d\delta \alpha ^\epsilon + \delta d\alpha ^\epsilon , \end{aligned}$$

A.2 $$\begin{aligned} \tau ^\epsilon= & {} \pi _H \tau ^\epsilon + d\delta \beta ^\epsilon + \delta d\beta ^\epsilon , \end{aligned}$$

where $\alpha ^\epsilon :=G(\omega ^\epsilon )$ and $\beta ^\epsilon :=G(\tau ^\epsilon )$. As before, $\pi _H: \Omega ^q(M)\mapsto \text {Har}^q (M)$ denotes the canonical projection onto the harmonic *q*-forms, and *G* denotes the Green operator for $\Delta$.

**3.** We now analyze the mode of convergence for each term in the Hodge decompositions of $\{\omega ^\epsilon \}$ and $\{\tau ^\epsilon \}$, i.e., Eqs. (A.1)–(A.2). We will summarize the results in the following two claims:

### Claim

In Eq. (A.1), $\{ \pi _H(\omega ^\epsilon )\}$ and $\{\delta d\alpha ^\epsilon \}$ are strongly convergent in $L^r(M;\bigwedge ^qT^*M)$, while the second term $\{d\delta \alpha ^\epsilon \}$ is weakly convergent in $L^r(M;\bigwedge ^q T^*M)$. Moreover, $\delta \alpha ^\epsilon$ is strongly convergent in $L^{r}(M;\bigwedge ^{q+1}T^*M)$.

Indeed, $\{\omega ^\epsilon \}$ is weakly convergent, hence bounded in $L^r$. On the other hand, $\pi _H \omega ^\epsilon \in \text {Har}^q(M)$, which is finite-dimensional, by Theorem 3.2. Then $\{\pi _H \omega ^\epsilon \}$ convergent strongly.

Next, we recall that *G* commutes with any operator commuting with $\Delta$; hence, $Gd=dG$ and $G\delta =\delta G$. Then we have

A.3 $$\begin{aligned} \delta d \alpha ^\epsilon = \delta \big (G (d\omega ^\epsilon )\big ). \end{aligned}$$

By assumption, $\{d\omega ^\epsilon \}$ is confined in a compact set $K_d \Subset W^{-1,r}_\mathrm{loc}(M;\bigwedge ^{q+1}T^*M)$ so that $\{\delta d\alpha ^\epsilon \}$ is strongly convergent, by the continuity of $\delta$ and *G*.

The middle term in decomposition (A.1) is a weakly convergent sequence, since $d\delta \alpha ^\epsilon = \omega ^\epsilon - \pi (\omega ^\epsilon )-\delta d\alpha ^\epsilon$. Moreover, we notice that $\delta \alpha ^\epsilon = G\delta \omega ^\epsilon$, which is bounded in $W^{1,r}(M;\bigwedge ^{q+1}T^*M)$, again by the rigidity of $\delta$ and *G*. Therefore, by the Rellich lemma, $\{\delta \alpha ^\epsilon \}$ is pre-compact in $L^r(M;\bigwedge ^{q+1}T^*M)$, hence converges strongly. This implies the claim.

Applying the similar arguments to Eq. (A.2), we can also verify

### Claim

In Eq. (A.2), $\{\pi _H \tau ^\epsilon \}$ and $\{d\delta \beta ^\epsilon \}$ are strongly convergent in $L^s(M;\bigwedge ^qT^*M)$, while $\{\delta d \beta ^\epsilon \}$ is weakly convergent in $L^s(M;\bigwedge ^qT^*M)$. Moreover, $\{d\beta ^\epsilon \}$ is strongly convergent in $W^{1,s}(M;\bigwedge ^{q-1}T^*M)$.

**4.** We are now at the stage for proving the convergence of $\langle \omega ^\epsilon ,\tau ^\epsilon \rangle$ in the distributional sense. In view of the Hodge decomposition in Eqs. (A.1)–(A.2), we have

A.4 $$\begin{aligned} \int _M \langle \omega ^\epsilon ,\tau ^\epsilon \rangle \psi \, \mathrm{d}V_g =&\left\{ \int _M \langle \pi _H \omega ^\epsilon , \pi _H\tau ^\epsilon \rangle \psi \,\mathrm{d}V_g + \int _M \langle \pi _H\omega ^\epsilon , d\delta \beta ^\epsilon \rangle \psi \,\mathrm{d}V_g \right. \nonumber \\&\,\,\,\,+\int _M \langle \pi _H\omega ^\epsilon , \delta d\beta ^\epsilon \rangle \psi \,\mathrm{d}V_g + \int _M \langle d\delta \alpha ^\epsilon , \pi _H\tau ^\epsilon \rangle \psi \,\mathrm{d}V_g \nonumber \\&\,\,\,\,+ \int _M \langle d\delta \alpha ^\epsilon , d\delta \beta ^\epsilon \rangle \psi \,\mathrm{d}V_g + \int _M \langle \delta d\alpha ^\epsilon , \pi _H\tau ^\epsilon \rangle \psi \,\mathrm{d}V_g \nonumber \\&\left. \,\,\,\, + \int _M \langle \delta d\alpha ^\epsilon ,d\delta \beta ^\epsilon \rangle \psi \,\mathrm{d}V_g + \int _M \langle \delta d\alpha ^\epsilon , \delta d\beta ^\epsilon \rangle \psi \,\mathrm{d}V_g\right\} \nonumber \\&\,\,\,\,+\int _M \langle d\delta \alpha ^\epsilon , \delta d \beta ^\epsilon \rangle \psi \,\mathrm{d}V_g, \end{aligned}$$

where each of the first eight terms in the bracket on the right-hand side is the pairing of one strongly convergent sequence and one weakly (or strongly) convergent sequence. This can be deduced immediately from the two claims in Step 3. Thus, as $\epsilon \rightarrow 0$, these eight terms pass to the desired limits, i.e.,

$$\begin{aligned} \int _M \langle \pi _H\omega ^\epsilon , \pi _H\tau ^\epsilon \rangle \psi \,\mathrm{d}V_g \rightarrow \int _M \langle \pi _H\bar{\omega }, \pi _H\bar{\tau } \rangle \psi \,\mathrm{d}V_g \qquad \hbox {as }\epsilon \rightarrow 0, \end{aligned}$$

and similarly for the other seven terms.

**5.** To deal with the last term on the right-hand side, which is a pairing of two weakly convergent sequences, we integrate by parts to find that

A.5 $$\begin{aligned} \int _M \langle d\delta \alpha ^\epsilon , \delta d \beta ^\epsilon \rangle \psi \,\mathrm{d}V_g =&\int _M d\big ( \psi \delta \alpha ^\epsilon \wedge *\delta d \beta ^\epsilon \big )\,\mathrm{d}V_g\nonumber \\&+(-1)^q \int _M \big (\delta \alpha ^\epsilon \wedge d(*\delta d\beta ^\epsilon )\psi \big )\,\mathrm{d}V_g \nonumber \\&+(-1)^n \int _M \big (\delta \alpha ^\epsilon \wedge *\delta d \beta ^\epsilon \wedge d\psi \big )\,\mathrm{d} V_g, \end{aligned}$$

where we have used the super-commutative property of the wedge product:

$$\begin{aligned} d(\omega \wedge \tau ) = d\omega \wedge \tau +(-1)^{\deg (\omega )}\omega \wedge d\tau . \end{aligned}$$

Now, in Eq. (A.5), the first term on the right-hand side vanishes by the Stokes’ theorem, and the second term vanishes because $$\delta =\pm *d *$$, $$**=\pm 1$$, and $$d\circ d=0$$ ($$*$$ denotes the Hodge star). For the remaining term, notice that, in Step 3, we have proved the boundedness of $$\{\delta \alpha ^\epsilon \}$$ in $$W^{1,r}(M;\bigwedge ^{q-1}T^*M)$$ so that, by the Rellich lemma, it strongly converges in $$L^r(M;\bigwedge ^{q-1}T^*M)$$; in addition, since $$\{\delta d\beta ^\epsilon \}$$ is weakly convergent in $$L^s(M;\bigwedge ^q T^*M)$$ and $$d\psi \in C^\infty (M;T^*M)$$, this term is the integral of the wedge product of one strongly convergent term and one weakly convergent term. Thus, as before, we can pass the limits to obtain that

$$\begin{aligned} \int _M \langle d\delta \alpha ^\epsilon , \delta d \beta ^\epsilon \rangle \psi \,\mathrm{d}V_g \rightarrow \int _M \langle d\delta \alpha , \delta d \beta \rangle \psi \,\mathrm{d}V_g \qquad \hbox {for any }\psi \in C^\infty _c(M). \end{aligned}$$

Therefore, in view of the decomposition in (A.4), we conclude

$$\begin{aligned} \int _M \langle \omega ^\epsilon , \tau ^\epsilon \rangle \psi \,\mathrm{d}V_g \longrightarrow \int _M \langle \overline{\omega }, \overline{\tau }\rangle \psi \,\mathrm{d}V_g \qquad \hbox {as }\epsilon \rightarrow 0. \end{aligned}$$

This completes the proof.$\square$
